# Fibro‐Adipogenic Progenitor Ablation Triggers Muscle Atrophy Through Cell Death‐Induced Inflammation

**DOI:** 10.1002/jcsm.70393

**Published:** 2026-09-29

**Authors:** Yangyi E. Luo, Young il Lee, Zoe Abe‐Teh, Rachel Y. Young, Lan Wei‐LaPierre, Elisabeth R. Barton

**Affiliations:** ^1^ Department of Applied Physiology and Kinesiology, College of Health and Human Performance University of Florida Gainesville Florida USA; ^2^ Myology Institute University of Florida Gainesville Florida USA; ^3^ Department of Pharmacology and Therapeutics, College of Medicine University of Florida Gainesville Florida USA; ^4^ Department of Computer and Information Science and Engineering, College of Engineering University of Florida Gainesville Florida USA

**Keywords:** CXC‐ligands, FAPs, inflammation, muscle atrophy, neuromuscular junction

## Abstract

**Background:**

Skeletal muscle mass maintenance involves coordination of myofibers with mononuclear cell populations, including satellite cells, resident macrophages and fibro‐adipogenic progenitors (FAPs). FAPs, identified by surface expression of PDGFRα, are important contributors to muscle homeostasis, as genetic ablation of FAPs induces rapid muscle atrophy. However, mechanisms underlying this response remain poorly understood.

**Methods:**

We utilized tamoxifen‐inducible FAP specific diptheria toxin (DTA) mice (*Pdgfra* ^Cre‐ERT2/+;^ *Rosa26* ^^DTA/+^) to evaluate consequences of FAP deletion, measuring body and muscle mass, and isolated function on the extensor digitorum longus (EDL) and soleus (SOL). Neuromuscular junction (NMJ) patency was assessed via whole‐mount staining, nerve vs. direct muscle stimulated function, and denervation‐responsive expression targeting AchRα/β/γ. Immunofluorescence of muscle sections was used to investigate cellular and morphological changes. RT‐qPCR measured expression of atrogenes, inflammatory chemokines/cytokines, and growth factors in tibialis anterior (TA) muscles. To modulate the immune response, mice were subjected to immune cell depletion, pharmacological Cxcr2 inhibition, or treatment with the anti‐inflammatory steroid Vamorolone (VBP15).

**Results:**

Following tamoxifen administration, FAP density decreased 90%, coupled with a 30% loss of lean mass (*p* < 0.001). Isolated contractile measurements showed that FAP‐deleted muscles exhibited 25% reductions in maximal tetanic force in the EDL and SOL, while specific force and vulnerability to contractile damage remained unchanged. Whole‐mount staining revealed preserved NMJ structural integrity, and functional testing showed no differences between nerve and direct muscle stimulation. RT‐qPCR showed no change in genes encoding AchR subunits. Longitudinal body composition tracking revealed that FAP‐deletion‐induced muscle and fat loss occurred during tamoxifen administration and coincided with a 10‐fold increase in the infiltration of macrophages and neutrophils. Robust 10‐ to 150‐fold increases (*p* < 0.0001) in chemokine transcript levels was observed soon after FAP deletion but preceded atrogene upregulation (*Trim63, Fbxo32, Sqstm1, Ulk1*). *Ccl2* increased ~80‐fold, *Ccl12* ~ 80‐fold, *Cxcl1* ~ 50‐fold, and *Cxcl2* ~ 50‐fold. While immune cell depletion exacerbated muscle atrophy by worsening mass loss and further increasing atrogene expression 5‐fold, Cxcr2 inhibition or VBP15 treatment restored muscle mass by 15% (*p* < 0.01). VBP15 treatment also resulted in a 50% reduction in atrogene expression and a 70% reduction in *Cxcl1/2* expression levels.

**Conclusions:**

Our findings demonstrate that FAP deletion results in simple muscle atrophy without affecting muscular contractile properties and NMJ function. The atrophy induced by the loss of FAPs occurs through an inflammation‐mediated, Cxcl1/2‐dependent mechanism, caused by the response to FAP cell death potentially coupled with the absence of FAP actions on the inflammatory environment.

## 1 Introduction

Skeletal muscle accounts for ~40% of total body mass and is indispensable for locomotion, physical performance, and metabolic homeostasis. Beyond its physiological functions, preservation of skeletal muscle mass is a major determinant of clinical outcomes in many pathological conditions, including ageing, cancer, and other chronic diseases [1].

Maintenance of muscle mass is governed by the dynamic balance between anabolic and catabolic processes regulating protein turnover [2]. Two principal proteolytic systems are activated during muscle wasting and contribute substantially to muscle protein loss [2]. Muscle‐enriched E3 ubiquitin ligases, such as MuRF1 (*Trim63*) and Atrogin‐1 (*Fbxo32*), are robustly induced under catabolic conditions, and their genetic ablation partially attenuates muscle loss [3]. Likewise, excessive autophagic activity has been implicated in the progression of muscle atrophy [4]. These catabolic pathways are regulated by upstream signalling networks, including the IGF‐I–Akt–mTOR axis [5, 6] and inflammatory signalling mediated by NF‐κB, a central transcriptional regulator of muscle wasting programs [7]. Despite substantial advances in defining the molecular mechanisms that govern muscle atrophy, less is known about the cellular sources and microenvironmental signals that initiate and sustain these catabolic responses.

Skeletal muscle multinucleated myofibers interact with a diverse network of resident mononuclear cells, including satellite cells (SCs) and fibro‐adipogenic progenitors (FAPs). These cells comprise 1%–4% and 5%–15% of the total nuclei in skeletal muscle, respectively [8, 9]. Early studies employing irradiation‐based models suggested that SCs were indispensable regulators of muscle adaptation [10]. However, the interpretation was complicated by irradiation effects on all resident cell populations. Subsequent genetic ablation studies demonstrated that while SCs are required for muscle regeneration, they are largely dispensable for load‐induced hypertrophy or disuse‐induced muscle atrophy [11, 12], prompting renewed interest in contributions of nonmyogenic stromal cells to muscle homeostasis.

Among these populations, FAPs have emerged as key regulators of skeletal muscle homeostasis. In addition to their contribution to fibrosis and fatty infiltration [13, 14], genetic deletion of FAPs has been shown to induce rapid muscle atrophy and impair postnatal growth [15, 16], and age‐associated declines in FAP abundance have been linked to progression of sarcopenia [17]. Despite their established importance, how FAPs preserve myofiber mass remain incompletely defined. For instance, prior work demonstrated that FAP deletion results in neuromuscular junction (NMJ) degeneration and denervation‐associated muscle atrophy ~3‐weeks postablation [16]. Other proposed mechanisms centre on the loss of FAP‐secreted factors, including insulin‐like growth factor I (IGF‐I) and follistatin (Fst). However, deletion of FAP‐derived IGF‐I fails to alter baseline muscle mass, and reductions in total muscle Fst occurs two weeks after the onset of acute atrophy [18, 19]. Consequently, these candidates either lack a standalone atrophic phenotype or manifest long after wasting occurs, implying the existence of alternative pathways that initiate early muscle loss. Further, while FAPs are responsive to mechanical stimuli and orchestrate extracellular matrix (ECM) remodelling [19, 20], it is unknown if FAP loss impairs muscle function. Thus, the goal of this study was to determine the proteolytic and inflammatory pathways that cause early phases of muscle atrophy using a transgenic mouse model affording inducible ablation of FAPs.

## 2 Methods

### 2.1 Animal Models

All animal protocols were approved by the University of Florida Institutional Animal Care and Use Committee. Mice were on a C57BL/6 background under a 12‐h light/dark cycle with ad libitum access to food and water. Inducible FAP‐KO mice were generated by crossing *Pdgfra* ^*Cre‐ERT2/+*^ (Jax#032770) [21] with *Rosa26* ^*DTA/DTA*^ mice (Jax#009669) [15]. Littermates retaining the *Rosa26* ^*DTA/DTA*^ allele but lacking *Pdgfra* ^*Cre‐ERT2*^ allele served as controls. Genotyping was performed using JAX‐specified primers and G2 Master Mix. To induce Cre recombination at 12–13 weeks of age, all mice received daily intraperitoneal (IP) injections of Tamoxifen (Tmx) dissolved in corn oil (20 mg/mL) at 100 μg/g body weight for 5 consecutive days. Vehicle only (corn oil) served as an injection control in both genotypes.

### 2.2 NMJ Preparations and Immunocytochemistry

Tissue preparation and fluorescent whole‐mount labelling of the NMJ and its components were performed as described previously [22]. Dissected extensor digitorum longus (EDL) and soleus (SOL) muscles were fixed in 4% paraformaldehyde (PFA) for 20 min at room temperature (RT) and rinsed with PBS. To label postsynaptic acetylcholine receptors (AChR), muscles were incubated with Alexa Fluor‐conjugated α‐bungarotoxin (α‐BTX; 0.1 mg/mL; Invitrogen). Presynaptic nerve terminals were labelled using a mixture of monoclonal antibodies against neurofilament and synaptic vesicles (2H3 and SV2; Developmental Studies Hybridoma Bank). Schwann cells and FAPs were identified using rabbit anti‐S100b (1:500; Dako) and goat anti‐PDGFRα (1:250; R&D Systems), respectively. Images were acquired with a Leica TPS II SP5 confocal microscope and analysed using FIJI/ImageJ software.

### 2.3 Targeted In Vivo Cell Depletion and Pharmacological Interventions

#### 2.3.1 Macrophage Depletion

Mice received an IP injection of 1 mg Clodrosome (clodronate liposomes) 2 days prior to the first tamoxifen dose. Controls received an equal volume of empty liposomes (Encapsome). Neutrophil depletion: Mice were administered IP injections of anti‐Ly6G antibody (Clone 1A8) over three intervals: 100 μg 1 day prior to Tmx, 50 μg after the second Tmx dose and 50 μg after the fourth Tmx dose. Controls received an equal volume of PBS. Cxcr2 inhibition: Mice received daily IP injections of the Cxcr2 antagonist SB‐225002 (10 μg/g in corn oil with 5% DMSO) starting 1 day prior to tamoxifen administration and continuing until tissue harvest. Controls received vehicle only. VBP‐15 (Vamorolone) treatment: Mice were administered daily oral doses of VBP‐15 (30 mg/kg) resuspended in cherry syrup starting 1 day prior to Tmx treatment. Controls received vehicle cherry syrup alone.

### 2.4 Additional Measurements

Detailed methods of ex vivo muscle contraction, tissue collection, immunofluorescence staining and RT‐qPCR are in the Supporting Information Methods.

## 3 Results

### 3.1 Deletion of FAPs Induces Acute, Fibre–Type‐Independent Skeletal Muscle Atrophy

We generated an inducible FAP ablation mouse model (FAP‐KO) and compared it to Tmx‐treated controls and vehicle only controls [15, 16]. Three weeks after Tmx administration (Figure 1a), FAP abundance was decreased by 90% in FAP‐KO muscles (Figure 1b). FAP deletion significantly reduced total body mass and lean mass in males and females, with a concomitant decrease in fat mass only in males (Figure 1b). Hindlimb muscles wet weight was significantly lower in tibialis anterior (TA), extensor digitorum longus (EDL) and soleus (SOL), accompanied by decreased myofiber cross‐sectional area (CSA, Figure 1c–f and Table S1). As these muscles have a range of metabolic and contractile properties, atrophy appeared independent of muscle type. Among other organs examined, spleens and livers in FAP‐KO mice were smaller by 30% and 17%, respectively. The inguinal fat pads also displayed decreases of 30% (Table S1). Neither vehicle treatment in both genotypes nor Tmx treatment in controls caused changes in any parameter. Collectively, inducible FAP ablation eliminated resident FAPs and induced a significant loss of lean mass and skeletal muscle size. The phenotype is consistent with prior studies [15, 16, 18], supporting a significant role for FAPs in adult skeletal muscle mass maintenance.

**FIGURE 1:**
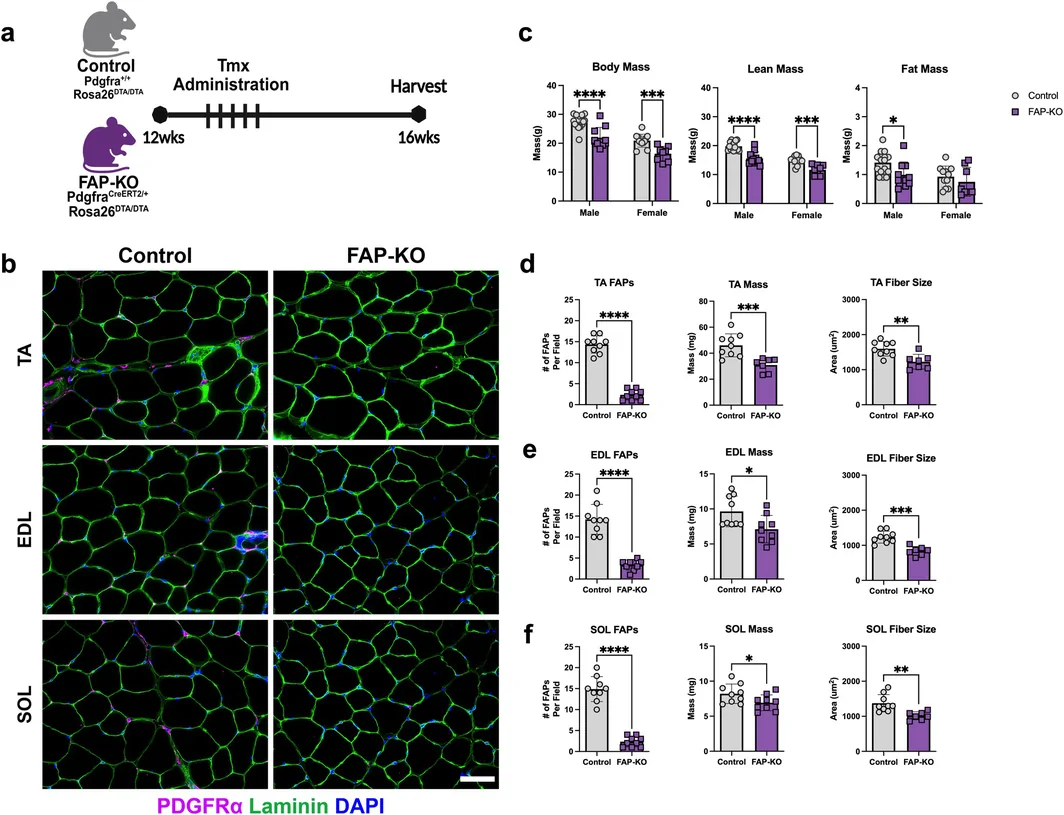
Inducible transgenic deletion of FAPs drives acute, fibre–type‐independent skeletal muscle atrophy. (a) Experimental schematic detailing the tamoxifen (Tmx) administration timeline in 12‐week‐old control and FAP‐KO mice, highlighting downstream tissue collection at 16 weeks of age. (b) Representative immunofluorescence images of Tibialis anterior (TA), Extensor digitorum longus (EDL) and Soleus (SOL) muscle cross‐sections from control and FAP‐KO mice, stained for PDGFRα (magenta), Laminin (green) and DAPI (blue) to visualize FAP deletion and myofiber boundaries. Scale bar, 50 μm. (c) Body composition analysis displaying total body mass, lean mass and fat mass in male and female cohorts, demonstrating significant global decreases in body and lean mass across both sexes, with fat mass loss restricted to males. *N* = 10 ~ 16 per sex per genotype. (d–f) Quantitative histological metrics and FAP clearance validation across metabolically diverse muscles: TA (D), EDL (E) and SOL (F). Each panel confirms an approximate 90% targeted reduction in interstitial FAPs alongside corresponding, robust reductions in absolute muscle wet weight and mean myofiber cross‐sectional area. *N* = 9 per genotype. Data are expressed as mean ± SD. Statistical significance was evaluated using an unpaired two‐tailed Student's *t*‐test, with male and female mice analysed separately. Asterisks (*) indicate significant differences between genotypes (*p < 0.05, **p < 0.01, ***p < 0.001, ****p < 0.0001).

### 3.2 FAPs Are Required for Absolute Force Production but Not Intrinsic Muscle Contractility

Although FAP ablation has been reported to reduce grip strength [15, 16], whether this reflects impaired function remains unclear. Absolute maximum isometric force in fast‐twitch EDL and slow‐twitch SOL muscles was significantly reduced in male and female FAP‐KO mice (Figure 2b,c), consistent with reduced muscle mass and fibre CSA (Figure 1c–f). However, normalization by physiological CSA revealed that specific force was unaltered in FAP‐KO mice compared to controls (Figure 2b,c). This indicates that intrinsic force generation capacity is retained and that short‐term FAP loss does not compromise muscle tissue quality. Ex vivo force–frequency relationships in FAP‐KO EDL and SOL muscles mirrored this absolute reduction without inducing a shift in the force–frequency profile (Figure 2d,e). To determine whether FAP deletion increases muscle fragility, we subjected EDL muscles to eccentric contractions [23, 24]. FAP‐KO muscles did not exhibit increased force deficits following five consecutive eccentric contractions compared with control EDLs in either sex (Figure 2f), indicating preserved myofiber structural integrity and force transmission after FAP loss. These data demonstrate that while acute, short‐term FAP deletion results in lower muscle force output, this is driven by quantitative reductions in mass rather than qualitative impairments of the contractile machinery.

**FIGURE 2:**
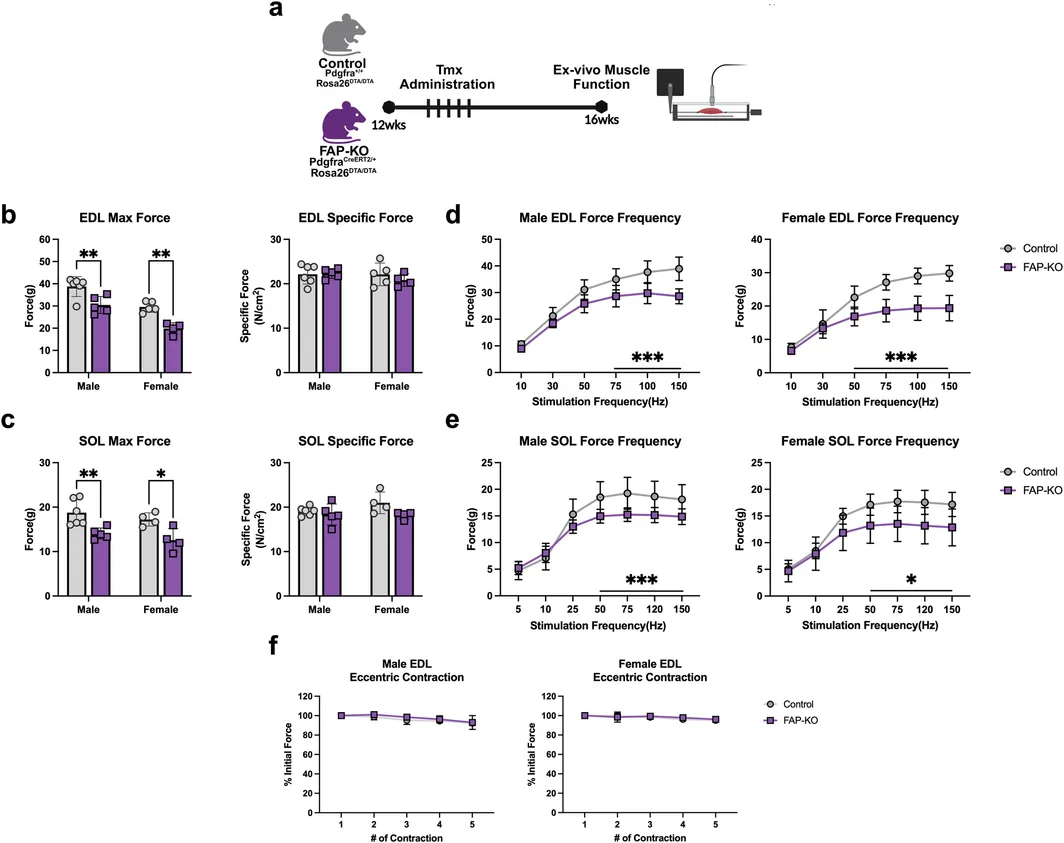
Loss of FAPs cause reduced absolute force production without a change in specific force or muscle fragility. (a) Experimental schematic detailing the tamoxifen (Tmx) administration timeline in 12‐week‐old control and FAP‐KO mice, highlighting downstream ex vivo muscle function analysis of the extensor digitorum longus (EDL) and soleus (SOL) muscles at 16 weeks of age. (b) Absolute maximum isometric force (g) and cross‐sectional area‐normalized specific force (N/cm^2^) of male and female EDL muscles, illustrating a significant deficit in absolute force capacity but preserved specific force. *N* = 4 ~ 6 per sex per genotype. (c) Absolute maximum isometric force (g) and normalized specific force (N/cm^2^) of male and female SOL muscles, illustrating a significant deficit in absolute force capacity but preserved specific force. *N* = 3 ~ 6 per sex per genotype. (d) Force–frequency relationships of male and female EDL muscles, highlighting a significant downward shift in absolute force production in FAP‐KO mice. *N* = 4 ~ 6 per sex per genotype. (e) Force–frequency relationships of male and female SOL muscles, highlighting a significant downward shift in absolute force production in FAP‐KO mice. *N* = 3 ~ 6 per sex per genotype. (f) Eccentric contraction profiles of male and female EDL muscles, expressed as a percentage of initial force across five repetitive lengthening contractions, demonstrating no significant alterations in contraction‐induced injury susceptibility between genotypes. *N* = 3 per sex per genotype. Data are expressed as mean ± SD. Statistical significance was evaluated using a two‐way ANOVA followed by Šidák's post hoc test. Asterisks (*) indicate significant differences between genotypes (*p < 0.05, **p < 0.01, ***p < 0.001).

### 3.3 Ablation of FAPs Does Not Alter Neuromuscular Junction Structure or Function

The localization of FAPs near the NMJ and their potential role in maintaining synaptic stability have been previously documented [17, 25], yet whether this has biological consequences at different ages of mice remains unknown. Whole‐mount immunofluorescence staining of NMJs was performed to evaluate synaptic architecture (Figure 3a). Despite the physical clustering of FAPs around the synaptic cleft, NMJ structure was unaltered in FAP‐KO EDL or SOL muscles (Figure 3b). Co‐labelling of presynaptic nerve terminals (Neurofilament) and postsynaptic acetylcholine receptors (AChRs; via α‐bungarotoxin, BTX) at the endplate showed 100% overlap between the presynaptic and postsynaptic apparatus (Figure 3b,c). BTX labelling outlined robust, intact postsynaptic gutters with no signs of fragmentation or degeneration [26]. Labelling of terminal Schwann cells using S100b antibodies revealed normal morphology and cellular distribution (Figure 3b). Collectively, these structural analyses demonstrate that acute FAP ablation does not induce structural denervation, postsynaptic disruption, or Schwann cell abnormalities.

**FIGURE 3:**
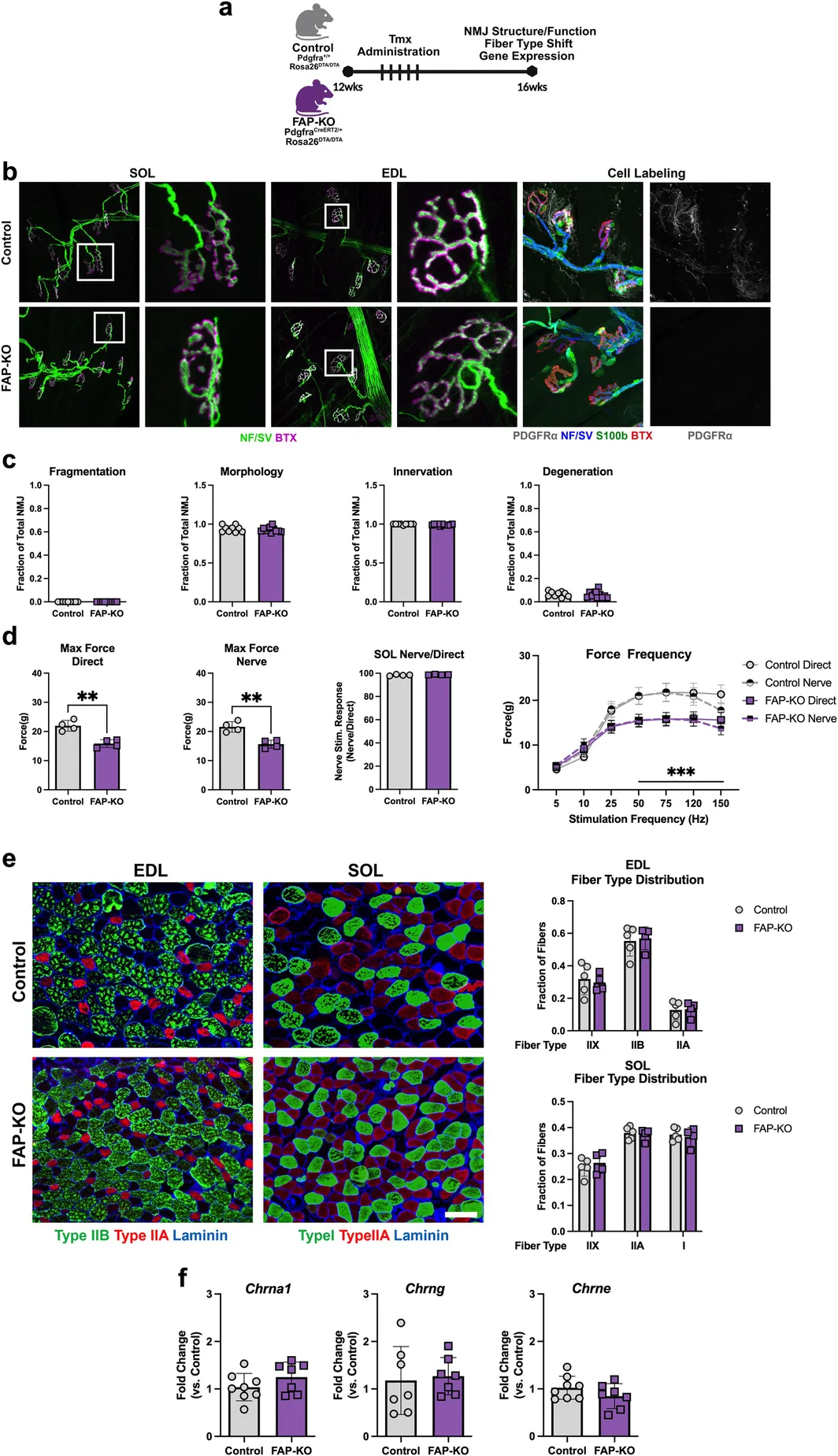
Structural and functional preservation of the neuromuscular junction (NMJ) following acute FAP deletion. (a) Experimental schematic detailing the tamoxifen (Tmx) administration timeline in 12‐week‐old Control and FAP‐KO mice, highlighting downstream multisystem analysis of NMJ structure/function, fibre‐type distribution and synaptic gene expression at 16 weeks of age. (b) Representative immunofluorescence confocal maximum intensity projection images of soleus (SOL) and extensor digitorum longus (EDL) neuromuscular junctions (NMJs) stained for neurofilament and synaptic vesicle proteins (NF/SV, green) and acetylcholine receptors (α‐bungarotoxin/BTX, magenta). Right column panels display high‐magnification cell labelling visualizing the spatial orientation of PDGFRα+ FAPs (grey), axons/terminals (NF/SV, blue), terminal Schwann cells (S100b, green), and postsynaptic endplates (BTX, red). Scale bar SV/NF (EDL, SOL) = 50 μm. Scale bar (cell labelling) = 20 μm. (c) Morphological quantification of NMJ features, including endplate fragmentation, overall structural morphology, innervation status and active degeneration metrics, demonstrating completely unaltered structural synaptic architecture between control and FAP‐KO groups. *N* = 9 per genotype. (d) Functional neuromuscular transmission assessments in the SOL muscle comparing direct muscle stimulation against indirect nerve stimulation, displaying equivalent deficits in maximum isometric force and force–frequency curves across both stimulation methods in FAP‐KO mice, with fully preserved nerve‐to‐direct response ratios (~100%). *N* = 4 per genotype. (e) Representative immunofluorescence cross‐sections and quantification of myofiber type distribution in EDL (Type IIB [green], Type IIA [red], Laminin [blue]) and SOL (Type I [green], Type IIA [red], Laminin [blue]) muscles, confirming that acute FAP clearance does not drive fibre‐type switching or selective fast/slow‐twitch atrophy. Scale bar = 100 μm. *N* = 4 ~ 6 per genotype. (f) Relative mRNA expression analysis (fold change vs. control) of postsynaptic nicotinic acetylcholine receptor subunits (*Chrna1*, *Chrng*, *Chrne*), indicating no compensatory transcriptional alterations or denervation‐like molecular signatures. *N* = 7 per genotype. Data are expressed as mean ± SD. Statistical significance was evaluated using a two‐way ANOVA followed by Šidák's post hoc test or an unpaired two‐tailed Student's *t*‐test where appropriate. Asterisks (*) indicate significant differences between genotypes (**p < 0.01, ***p < 0.001).

To further determine whether NMJs in FAP‐KO mice are also functionally intact, we isolated SOL muscles from control and FAP‐KO mice with tibia nerves attached. Direct comparison of isometric force elicited via nerve stimulation versus direct muscle stimulation serves as an indicator of NMJ transmission [23]. Consistent with our direct muscle stimulation data (Figure 2), FAP‐KO muscles exhibited a significant reduction in absolute maximum force under both direct and indirect stimulation cascades (Figure 3d), and no difference in isometric force between direct muscle stimulation and nerve‐evoked contractions was detected (Figure 3d). Force–frequency relationships across maximal and submaximal stimulation frequencies further confirmed this parallel decline, showing no transmission failure at the NMJ in FAP‐KO SOL muscles (Figure 3d).

Another indicator for muscle denervation is fibre‐type transition. Therefore, we evaluated myofiber type distribution in the EDL and SOL (Figure 3e) muscles in FAP‐KO and control mice. Despite the pronounced reduction in myofiber cross‐sectional area (Figure 1), the relative distribution and percentages of slow and fast fibre types remained unaltered in both muscles (Figure 3e). Finally, we assessed transcription of genes encoding AChR subunits, as the upregulation of *Chrng* and downegulation of *Chrne* serve as classic molecular signatures of denervation [24, 27]. RT‐qPCR demonstrated that AChR subunit expression remained unchanged between control and FAP‐KO cohorts (Figure 3f).

Taken together, structural, functional and molecular analyses demonstrate that while acute FAP deletion drives rapid muscle atrophy, the neuromuscular apparatus remains structurally and functionally intact during the window examined in this study. This evidence strongly argues that loss of FAPs causes skeletal muscle wasting through an NMJ‐independent mechanism.

### 3.4 The Timecourse of Atrophy After FAP Deletion Is Associated With Innate Immune Cell Infiltration

Our initial cohorts were analysed 28‐day post‐Tmx (dpt) to allow for a full washout period and to prevent confounding vehicle or drug effects [19, 28]. However, because Cre‐recombination and subsequent DTA expression in PDGFRα+ cells initiate cell death immediately upon Tmx exposure, we sought to capture the dynamics of wasting concurrent with FAP deletion. Longitudinal body composition measurements revealed that most of the lean mass loss occurred during the active Tmx administration phase, rather than in the subsequent washout period (Figure 4a,b). Fat mass followed a similar trajectory, with ~30% reduction observed 5 dpt in FAP‐KO mice (Figure S1a). The acute dosing window was a critical phase governing the atrophic and catabolic processes. To determine if mass loss was due to reduced appetite, food and water consumption was monitored prior to and throughout 5 days of Tmx or oil treatment. Food intake was lower in the first 2 days of Tmx treatment in both genotypes, and water intake was increased in the same groups 5 days after the start of treatment (Figure S2). However, these changes were independent of the loss of FAPs. Subsequent evaluations were restricted to the TA muscle, which represented the atrophy responses of all muscles analysed. Characterization of the TA at 1, 3 and 5 dpt revealed a progressive decline in muscle wet weight in FAP‐KO compared to controls (Figure 4c). This reduction was significant by 5 dpt. These data indicate that FAP deletion‐driven atrophy is an acute process initiated by the third Tmx dose.

**FIGURE 4:**
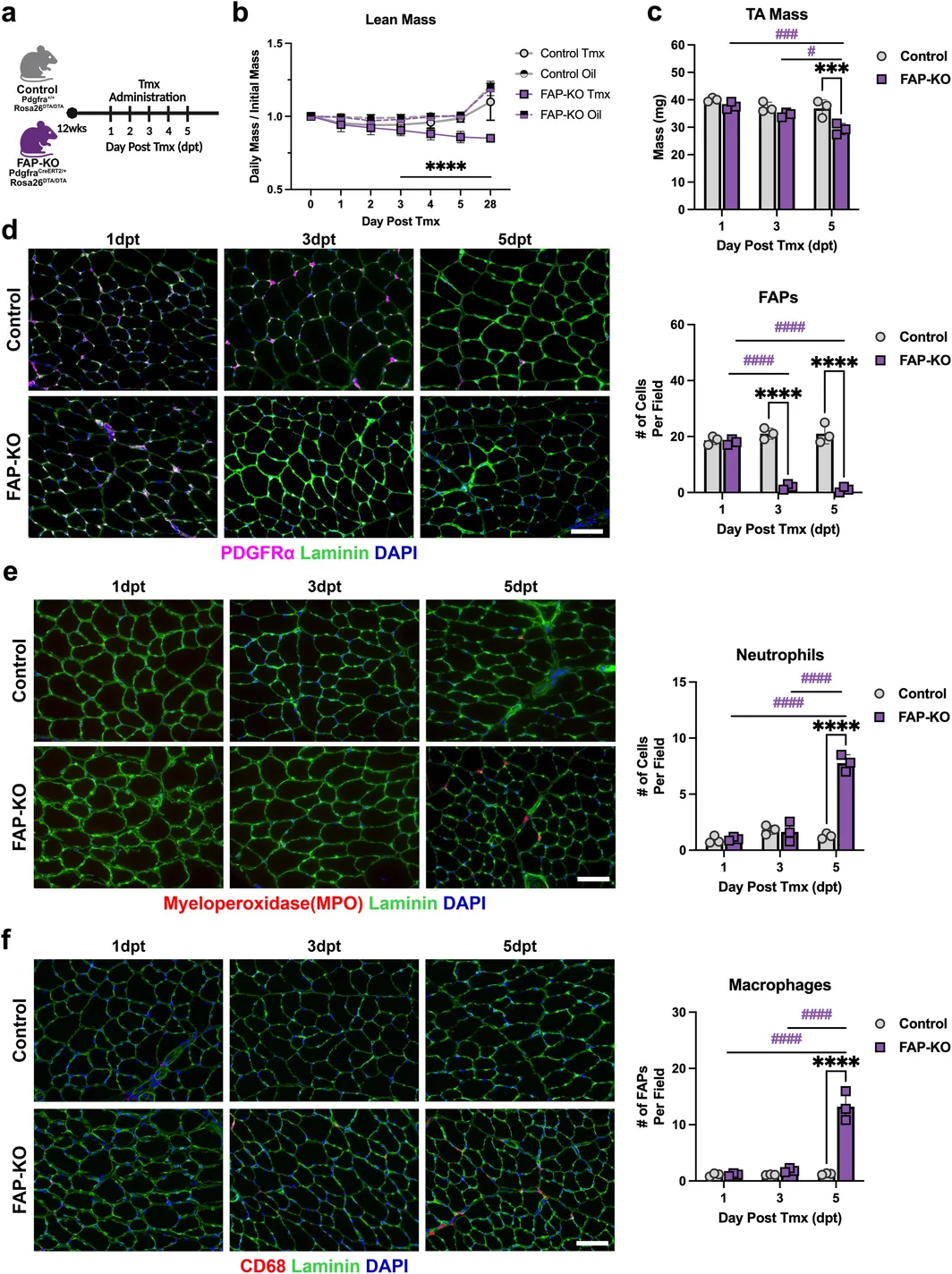
Acute kinetics of FAP deletion reveal rapid muscle wasting coupled with innate immune cell infiltration. (a) Experimental schematic detailing the short‐term tamoxifen (Tmx) administration timeline (Days 1–5) in 12‐week‐old Control and FAP‐KO mice, highlighting acute downstream tissue harvest time points at 1‐, 3‐, 5‐ and 28‐day post‐Tmx initiation (dpt). (b) Longitudinal lean mass tracking normalized to initial lean mass, demonstrating a rapid and significant divergence and progressive loss of lean mass in FAP‐KO mice starting at 3 dpt. *N* = 25 ~ 28 per genotype. (c) Absolute Tibialis Anterior (TA) muscle mass across acute time points, illustrating a significant reduction in absolute muscle mass by 5 dpt. *N* = 3 per timepoint per genotype. Two‐way ANOVA: genotype *p* = 0.0006, timepoint *p* = 0.0005. (d) Representative immunofluorescence cross‐sections and corresponding quantification of resident FAPs stained for PDGFRα (magenta), Laminin (green) and DAPI (blue), confirming rapid and near‐complete interstitial FAP clearance by 3 dpt. Scale bar = 100 μm. *N* = 3 per timepoint per genotype. Two‐way ANOVA: genotype *p* < 0.0001, timepoint *p* < 0.0001. (e) Representative immunofluorescence cross‐sections and corresponding quantification of infiltrating neutrophils stained for Myeloperoxidase (MPO, red), Laminin (green) and DAPI (blue), highlighting a massive, synchronized influx of neutrophils at 5 dpt. Scale bar = 100 μm. *N* = 3 per timepoint per genotype. Two‐way ANOVA: genotype *p* < 0.0001, timepoint *p* < 0.0001. (f) Representative immunofluorescence cross‐sections and corresponding quantification of infiltrating macrophages stained for CD68 (red), Laminin (green) and DAPI (blue), revealing a robust, parallel accumulation of interstitial macrophages peaking at 5 dpt. Scale bar = 100 μm. *N* = 3 per timepoint per genotype. Two‐way ANOVA: genotype *p* < 0.0001, timepoint *p* < 0.0001.Data are expressed as mean ± SD. Statistical significance was evaluated using a two‐way ANOVA followed by Šidák's post hoc test. Asterisks (*) indicate significant differences between genotypes at a specific time point (***p < 0.001, ****p < 0.0001). Pound signs (#) indicate significant differences across time points within the same genotype (#*p* < 0.05, ###*p* < 0.001, ####*p* < 0.0001).

Given that our model introduced cell death in vivo, we hypothesized that the sudden disruption of FAPs provoked a local inflammatory response. To map these cellular dynamics, we performed immunofluorescence staining to track FAPs, myeloperoxidase‐positive (MPO+) neutrophils and CD68+ macrophages [25, 26]. Apoptotic FAPs identified by cleaved caspase 3 were detectable at 1 dpt (Figure S3a,b), and a significant reduction in FAP abundance was observed at 3 dpt (Figure 4d). The recruitment of innate immune cells exhibited a temporal lag, with marked expansion and infiltration of neutrophils and macrophages not observed until 5 dpt (Figure 4e,f). This temporal sequencing suggests that the robust immune response at 5 dpt is a reaction to the initial wave of FAP cell death triggered by 1–3 dpt and that infiltration of early responding immune cells is a critical event during FAP deletion induced immune clearance.

### 3.5 Temporal Profiling Links Early Chemokine Expression to Atrogene Activation

To investigate the downstream consequences of acute FAP deletion and the subsequent innate immune infiltration, we performed RT‐qPCR on whole muscle cDNA during the initial stages of ablation (1, 3 and 5 dpt) compared to pre‐Tmx treatment and 28 dpt. We tracked the relationship between transcriptional alterations in atrophying myofibers and the activation of key inflammatory cascades (Figure 5c–e). Target genes were categorized into three main groups: atrogenes (*Trim63, Fbxo32, Ulk1* and *Sqstm1*) [23, 24], inflammatory chemokines/cytokines (C‐C and C‐X‐C motif ligands) and classic pro‐inflammatory cytokines of the interleukin family, known to drive muscle catabolism [27, 29].

**FIGURE 5:**
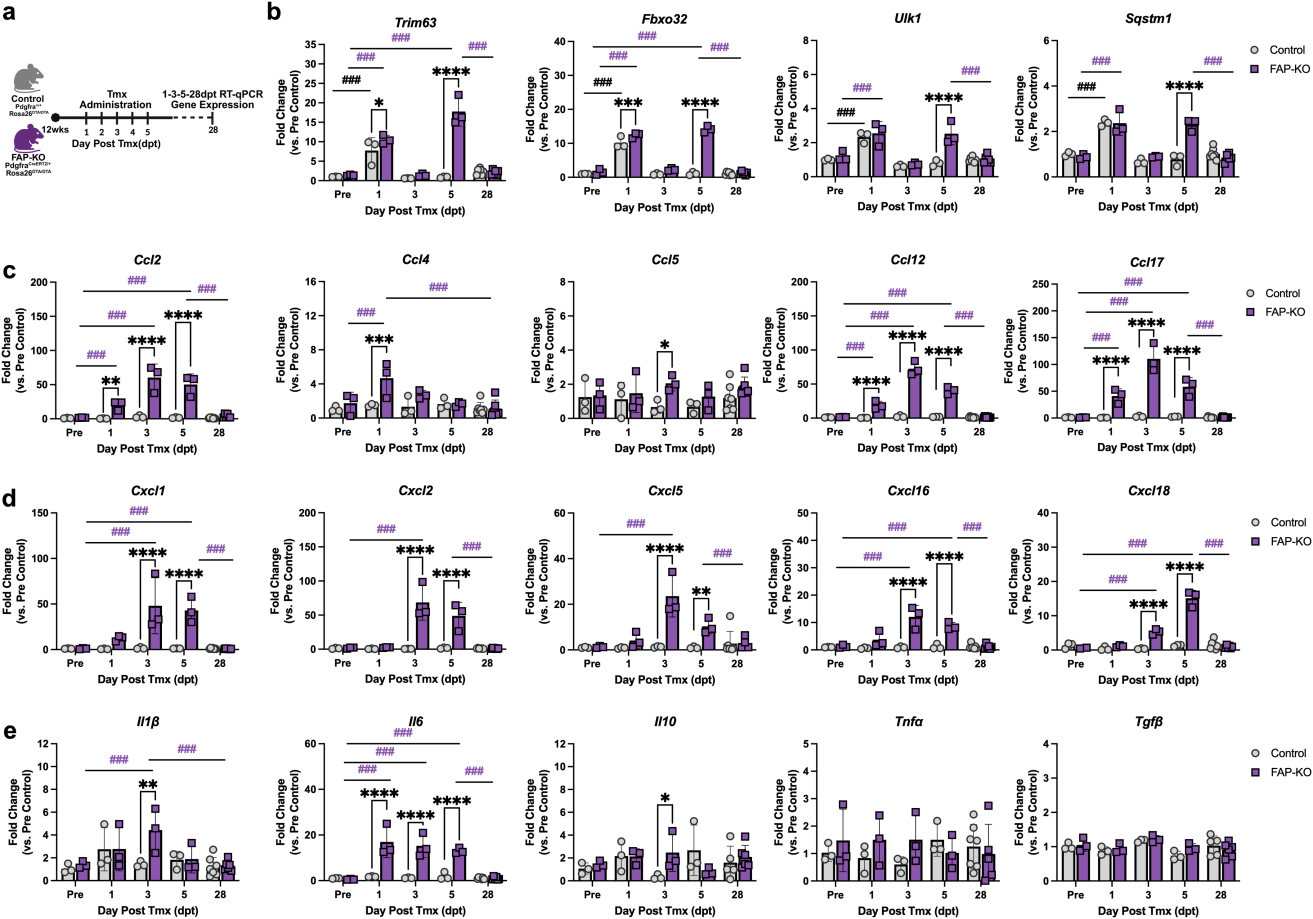
Temporal transcriptional dynamics of ubiquitin‐proteasome, autophagic and inflammatory pathways during acute FAP deletion. (a) Experimental schematic detailing the short‐term tamoxifen (Tmx) administration timeline in 12‐week‐old Control and FAP‐KO mice, highlighting the temporal framework for downstream gastrocnemius muscle collection and RT‐qPCR analysis at pre‐, 1‐, 3‐, 5‐ and 28‐day post‐tamoxifen initiation (dpt). (b) Relative mRNA expression analysis (fold change vs. Pre Control) of primary muscle‐specific E3 ubiquitin ligases (*Trim63*, *Fbxo32*) and core autophagy‐lysosomal markers (*Ulk1*, *Sqstm1*), revealing a rapid initial upregulation at 1 dpt and a secondary, coordinated proteolytic surge at 5 dpt that completely returns to baseline by 28 dpt. *N* = 3 ~ 7 per timepoint per genotype. *Trim63*: Two‐way ANOVA: genotype *p* < 0.0001, timepoint *p* < 0.0001. *Fbxo32*: Two‐way ANOVA: genotype *p* < 0.0001, timepoint *p* < 0.0001. *Ulk1*: Two‐way ANOVA: genotype *p* < 0.0001, timepoint *p* = 0.0003. *Sqstm1*: Two‐way ANOVA: genotype *p* < 0.0001, timepoint *p* = 0.0011. (c) Relative mRNA expression profiling of CC‐chemokine ligands (*Ccl2*, *Ccl4*, *Ccl5*, *Ccl12*, *Ccl17*), demonstrating an early, robust and sustained transcriptional activation that peaks between 1 dpt and 3 dpt in FAP‐KO tissue. *N* = 3 ~ 7 per timepoint per genotype. *Ccl2*: Two‐way ANOVA: genotype *p* < 0.0001, timepoint *p* < 0.0001. *Ccl4*: Two‐way ANOVA: genotype *p* = 0.0046, timepoint *p* = 0.0035. *Ccl5*: Two‐way ANOVA: genotype *p* = 0.0233, timepoint *p* = 0.6863. *Ccl12*: Two‐way ANOVA: genotype *p* < 0.0001, timepoint *p* < 0.0001. *Ccl17*: Two‐way ANOVA: genotype *p* < 0.0001, timepoint *p* < 0.0001. (d) Relative mRNA expression profiling of CXC‐chemokine ligands (*Cxcl1*, *Cxcl2*, *Cxcl5*, *Cxcl16*, *Cxcl18),* showcasing a highly synchronized, massive transcriptional surge that heavily concentrates within the 3 and 5 dpt windows. *N* = 3 ~ 7 per timepoint per genotype. *Cxcl1*: Two‐way ANOVA: genotype *p* < 0.0001, timepoint *p* < 0.0001. *Cxcl2*: Two‐way ANOVA: genotype *p* < 0.0001, timepoint *p* < 0.0001. *Cxcl5*: Two‐way ANOVA: genotype *p* < 0.0001, timepoint *p* = 0.0003. *Cxcl16*: Two‐way ANOVA: genotype *p* < 0.0001, timepoint *p* < 0.0001. *Cxcl18*: Two‐way ANOVA: genotype *p* < 0.0001, timepoint *p* < 0.0001. (e) Relative mRNA expression tracking of canonical systemic cytokines (*Il1β*, *Il6*, *Il10*, *Tnfa*, *Tgfb*), highlighting a profound and persistent elevation of Il6 alongside localized *Il1β* and *Il10* expression spikes at 3 dpt, with no significant alterations detected in *Tnfa* or *Tgfb* expression. *N* = 3 ~ 7 per timepoint per genotype. *Il1b*: Two‐way ANOVA: genotype *p* = 0.0713, timepoint *p* = 0.011. *Il‐6*: Two‐way ANOVA: genotype *p* < 0.0001, timepoint *p* < 0.0001. *Il‐10*: Two‐way ANOVA: genotype *p* = 0.6733, timepoint *p* = 0.6. *Tnfa*: Two‐way ANOVA: genotype *p* = 0.3729, timepoint *p* = 0.9886. *Tgfb*: Two‐way ANOVA: genotype *p* = 0.3895, timepoint *p* = 0.0233. Data are expressed as mean ± SD Statistical significance was evaluated using a two‐way ANOVA followed by Šidák's post hoc test. Asterisks (*) indicate significant differences between genotypes at a specific time point (*p < 0.05, **p < 0.01, ***p < 0.001, ****p < 0.0001). Pound signs (#) indicate significant differences across time points within the same genotype (###*p* < 0.001).

The atrogene panel exhibited dynamic expression profiles during the early window of atrophy. At 1 dpt, we observed a transient, significant upregulation of *Trim63*, *Fbxo32*, *Ulk1* and *Sqstm1* in FAP‐KO and control groups (Figure 5b). Because this activation occurred regardless of genotype, this initial response likely represents transient stress induced by exogenous Tmx administration. Following this initial spike, atrogene expression returned to baseline levels in control and FAP‐KO muscles at 3 dpt. However, only FAP‐KO muscles exhibited a secondary surge in atrogene expression at 5 dpt (Figure 5b). This late‐stage activation supports a specific, FAP deletion‐driven induction of proteolytic and autophagic machinery. Upregulated atrogene expression returned to baseline levels by 28 dpt. To determine whether the acute muscle atrophy induced by FAP deletion was driven by the loss of FAP‐derived trophic factors, we examined skeletal muscle transcript levels of *Igf1, Fst, Hgf* and *Fgf2* (Figure S4a–d) [18]. Among these factors, a transient 75% reduction in *Igf1* was evident at 5 dpt in FAP‐KO muscles, with a twofold decrease in *Fst* expression found at 28 dpt (Figure S4a,b). Reduced *Igf1* expression aligns with FAPs serving as a significant IGF‐I source, but its targeted deletion from FAPs did not cause atrophy [19]. Further, lower *Fst* expression does not align with the initial atrophy. Measurements of *Mstn* expression were also performed, which exhibited significant increases in both genotypes 1 and 3 dpt, with further increases in FAP‐KO muscles 5 dpt (Figure S4e). These findings suggest that acute alterations in IGF‐I and myostatin levels may contribute to acute muscle atrophy.

Next, to determine if this myofiber‐intrinsic proteolysis was coupled to upstream inflammatory signalling, we profiled a targeted panel of chemokines primarily secreted by active myeloid cells. Our analysis revealed a global induction of inflammatory transcripts only within FAP‐KO muscles, though individual ligands exhibited distinct temporal dynamics (Figure 5c,d). Among these, *Ccl2*, *Ccl12*, *Cxcl1* and *Cxcl2* were identified as the primary drivers of this inflammatory microenvironment, with 50‐to‐100‐fold increases in FAP‐KO muscles compared to controls (Figure 5c,d). Chemokine expression peaked sharply at 3 dpt, preceding the secondary surge of atrogenes observed at 5 dpt. This tight temporal sequencing implied that early interstitial chemokine production may act as an inductive upstream signal driving subsequent myofiber proteolysis. Other chemokines, including *Ccl4, Ccl5, Cxcl5* and *Cxcl16*, demonstrated significant but less robust responses, showing 5‐to‐10‐fold increases across the time course (Figure 5d). This indicates that these specific ligands may play different roles in general immune cell clearance and homeostasis rather than driving the central hyper‐inflammatory cascade [30, 31]. We next evaluated expression of classic upstream pro‐inflammatory cytokines (*Il1b, Il6, Il10* and *Tnfa*). *Il6* was the only cytokine to show substantial induction, displaying ~15‐fold increase that was initiated early at 1 dpt and sustained through 5 dpt (Figure 5e). Minimal alterations in *Tnfa* and *Tgfb* expression were observed (Figure 5e). To determine whether the inflammatory cytokines were also observed in the circulation, we measured IL‐6 serum levels, a commonly used marker of systemic inflammation [32]. Despite the pronounced inflammatory response observed within skeletal muscle, only modest elevation in circulating IL‐6 occurred in FAP‐KO mice (Figure S4f), suggesting that FAP deletion elicits a predominantly local inflammatory response with minimal systemic inflammation.

Overall, this surge of local inflammation is restricted to the early stage of FAP deletion, returning to the baseline at 28 dpt, which indicates that deletion of FAPs stimulates an acute muscle loss instead of a chronic or progressive muscle wasting condition (Figure 5b–e). In addition, this transcriptional signature diverges sharply from the classic cytokine profiles documented during early‐stage muscle injury and regeneration [33], indicating that the cellular response to acute FAP deletion represents a nonregenerative inflammatory phenotype that directly primes the muscle for atrophy.

### 3.6 Myeloid Cell Depletion Exacerbates Atrophy and Disrupts Basal Muscle Homeostasis

To evaluate the involvement of infiltrating innate immune cells during the early phase of FAP deletion, we performed targeted chemical and antibody‐mediated depletion of macrophages, neutrophils or both populations combined, 1 day prior and during Tmx administration. Body composition tracking through 5 dpt demonstrated that macrophage depletion, neutrophil depletion and simultaneous double depletion all significantly exacerbated lean mass loss during FAP ablation (Figure 6b), but they did not cause more fat mass loss (Figure S1b–d). Since most of the lean muscle loss occurred at 5 dpt with elevated atrogene and chemokine expression, our downstream experiments focused on 5 dpt. The efficiency and specificity of these strategies were verified via immunofluorescence staining, confirming a robust reduction in their respective myeloid cell markers at 5 dpt (Figure 6c,d). Neutrophil depletion resulted in an increased population of macrophages; similarly, macrophage depletion led to an increase in neutrophils (Figure 6d). Intriguingly, the systemic clearance of these immune cells also induced significant muscle atrophy within control muscles (Figure 6d). These data imply that immune cells are required to maintain normal tissue homeostasis and protect muscle integrity during acute stress, likely by executing critical immune clearance and resolving cellular debris. Alternatively, the increased presence of dying cells may also have worsened the atrophy response.

**FIGURE 6:**
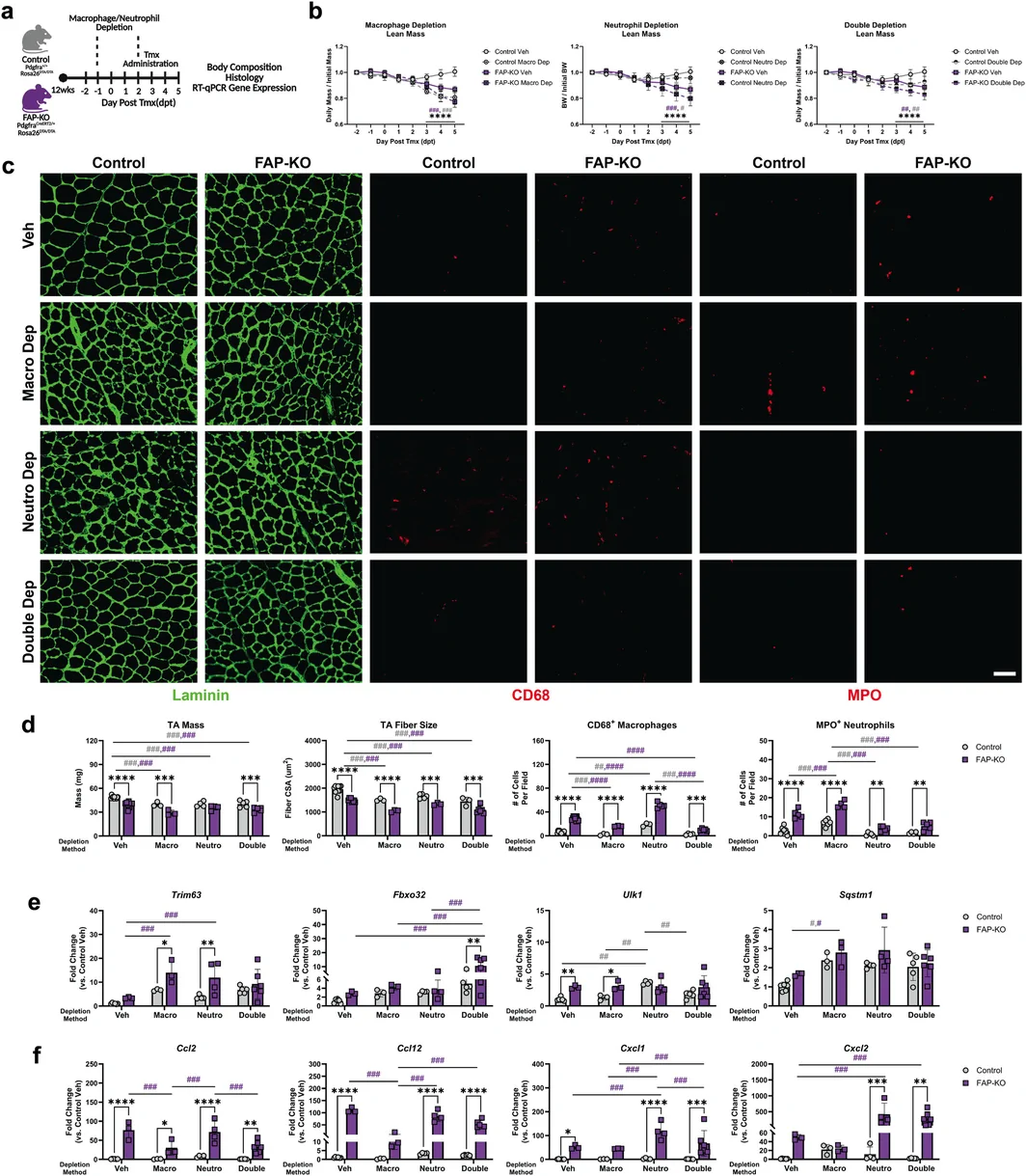
Systemic myeloid cell depletion accelerates muscle wasting and differentially dysregulates atrogene and chemokine transcription networks during acute FAP deletion. (a) Experimental schematic detailing the timeline for targeted macrophage and/or neutrophil depletion strategies initiated prior to tamoxifen (Tmx)‐induced FAP deletion, with downstream endpoints tracking body composition, histology and transcriptomic alterations. (b) Longitudinal tracking of normalized daily lean mass or body weight across macrophage depletion, neutrophil depletion and combined double depletion cohorts, demonstrating that physical clearance of these myeloid subsets significantly accelerates or worsens wasting kinetics. *N* = 4 ~ 6 per timepoint per genotype per treatment. Macrophage depletion: Two‐way ANOVA: genotype *p* < 0.0001, treatment *p* < 0.0001. Neutrophil depletion: Two‐way ANOVA: genotype *p* < 0.0001, treatment *p* < 0.0001. Double depletion: Two‐way ANOVA: genotype *p* < 0.0001, treatment *p* < 0.0001. (c) Representative immunofluorescence muscle cross‐sections across vehicle (Veh), macrophage‐depleted (Macro Dep), neutrophil‐depleted (Neutro Dep) and double‐depleted (Double Dep) cohorts, stained for Laminin (green), CD68 (red, macrophage marker) and MPO (red, neutrophil marker) to validate specific immunodepletion within the interstitial spaces at 5 dpt. Scale bar = 100 μm. (d) Quantitative metrics assessing absolute Tibialis Anterior (TA) muscle wet weight, mean myofiber cross‐sectional area (Fibre CSA) and the spatial abundance of interstitial CD68+ macrophages and MPO+ neutrophils per high‐power field, validating experimental cell depletions alongside un‐rescued myofiber atrophy metrics at 5 dpt. TA Mass: Two‐way ANOVA: genotype *p* < 0.0001, treatment *p* < 0.0001. TA fibre size: Two‐way ANOVA: genotype *p* < 0.0001, treatment *p* < 0.0001. CD68 macrophage: Two‐way ANOVA: genotype *p* < 0.0001, treatment *p* < 0.0001. MPO Neutrophil: Two‐way ANOVA: genotype *p* < 0.0001, treatment *p* < 0.0001. (e) Relative mRNA expression analysis (fold change vs. Control Veh) of canonical muscle‐specific E3 ubiquitin ligases (*Trim63, Fbxo32*) and key autophagy‐lysosomal markers (*Ulk1, Sqstm1*), showing heightened baseline atrogene induction upon myeloid cell removal at 5 dpt. *Trim63*: Two‐way ANOVA: genotype *p* = 0.0032, treatment *p* = 0.001. *Fbxo32*: Two‐way ANOVA: genotype *p* = 0.0316, treatment *p* = 0.0008. *Ulk1*: Two‐way ANOVA: genotype *p* = 0.0032, treatment *p* = 0.0394. *Sqstm1*: Two‐way ANOVA: genotype *p* = 0.0322, treatment *p* = 0.0026. (f) Relative mRNA expression profiling of downstream CC‐chemokines (*Ccl2*, *Ccl12*) and CXC‐chemokines (*Cxcl1*, *Cxcl2*) within the whole‐muscle local tissue niche across the various cellular depletion cohorts at 5 dpt. *Ccl2*: Two‐way ANOVA: genotype *p* < 0.0001, treatment *p* = 0.0016. *Ccl12*: Two‐way ANOVA: genotype *p* < 0.0001, treatment *p* < 0.0001. *Cxcl1*: Two‐way ANOVA: genotype *p* < 0.0001, treatment *p* = 0.0426. *Cxcl2*: Two‐way ANOVA: genotype *p* = 0.0019, treatment *p* = 0.04. Data are expressed as mean ± SD. Statistical significance was evaluated using a two‐way ANOVA followed by Tukey's post hoc test for multiple comparisons. Asterisks (*) indicate significant differences between genotypes under the same depletion condition (*p < 0.05, **p < 0.01, ***p < 0.001, ****p < 0.0001). Pound signs (#) indicate significant differences between depletion methods or experimental treatment groups (#*p* < 0.05, ##*p* < 0.01, ###*p* < 0.001, ####*p* < 0.0001).

Next, we performed RT‐qPCR analysis at 5 dpt to determine how the absence of specific myeloid subsets altered the expression of atrogenes and the chemokines that were most affected in FAP‐KO muscles. Consistent with the more extreme muscle atrophy, immune cell depletion further elevated the expression of the E3 ubiquitin ligases *Trim63* and *Fbxo32* in FAP‐KO muscles, with a similar upward atrophic trend in the control groups (Figure 6e). Double depletion did not cause an additive increase in *Trim63* expression but dramatically increased *Fbxo32* transcription. In contrast, the autophagy‐lysosomal markers *Ulk1* and *Sqstm1* displayed a substantially milder induction under immune cell depletion conditions (Figure 6e). This phenotypic divergence suggests that distinct intramuscular catabolic pathways are differentially activated depending on which cellular components are absent from the interstitium.

We focused on the four chemokines previously identified as hyper‐reactive to track how the local inflammatory profile shifted at 5 dpt. Selective macrophage depletion significantly blunted *Ccl2* and *Ccl12* expression within the muscle tissue (Figure 6f), suggesting that macrophages were the source *Ccl2* and *Ccl12*. However, this reduction in *Ccl2* and *Ccl12* levels failed to rescue muscle mass, myofiber size or elevated atrogene expression. In contrast, the selective ablation of neutrophils further compounded the hyper‐inflammatory microenvironment, exhibiting no change in *Ccl2* and *Ccl12* induction compared with nondepleted muscle, while provoking a higher elevation of *Cxcl1* and *Cxcl2* expression (Figure 6f). Collectively, this indicates that elevated expression of *Cxcl1* and *Cxcl2* correlates directly with heightened atrogene expression and muscle wasting, highlighting a complex, protective role for balanced innate immune infiltration during acute progenitor cell clearance.

### 3.7 Pharmacological Targeting of NF‐κB and the Cxcr2 Axis Ameliorates Muscle Wasting Associated With FAP Deletion

Because immune cell depletion highlighted a potential role of Cxcl1 and Cxcl2 in FAP ablation‐induced muscle atrophy, we investigated whether mitigating the downstream hyper‐inflammatory cascade—without clearing the cells themselves—could alleviate muscle wasting. Mice were treated with VBP15 (Vamorolone), the dissociative anti‐inflammatory steroid and NF‐κB inhibitor [3] or the selective Cxcr2 antagonist SB225002, which directly inhibits the hyper‐reactive Cxcl1/2 signalling axis, 2 days prior to Tmx administration (Figure 7a). Longitudinal body composition tracking revealed a significant rescue of lean mass and fat mass loss in FAP‐KO mice under both therapeutic regimens, while displaying negligible impacts on control cohorts (Figures 7b and S1e,f).

**FIGURE 7:**
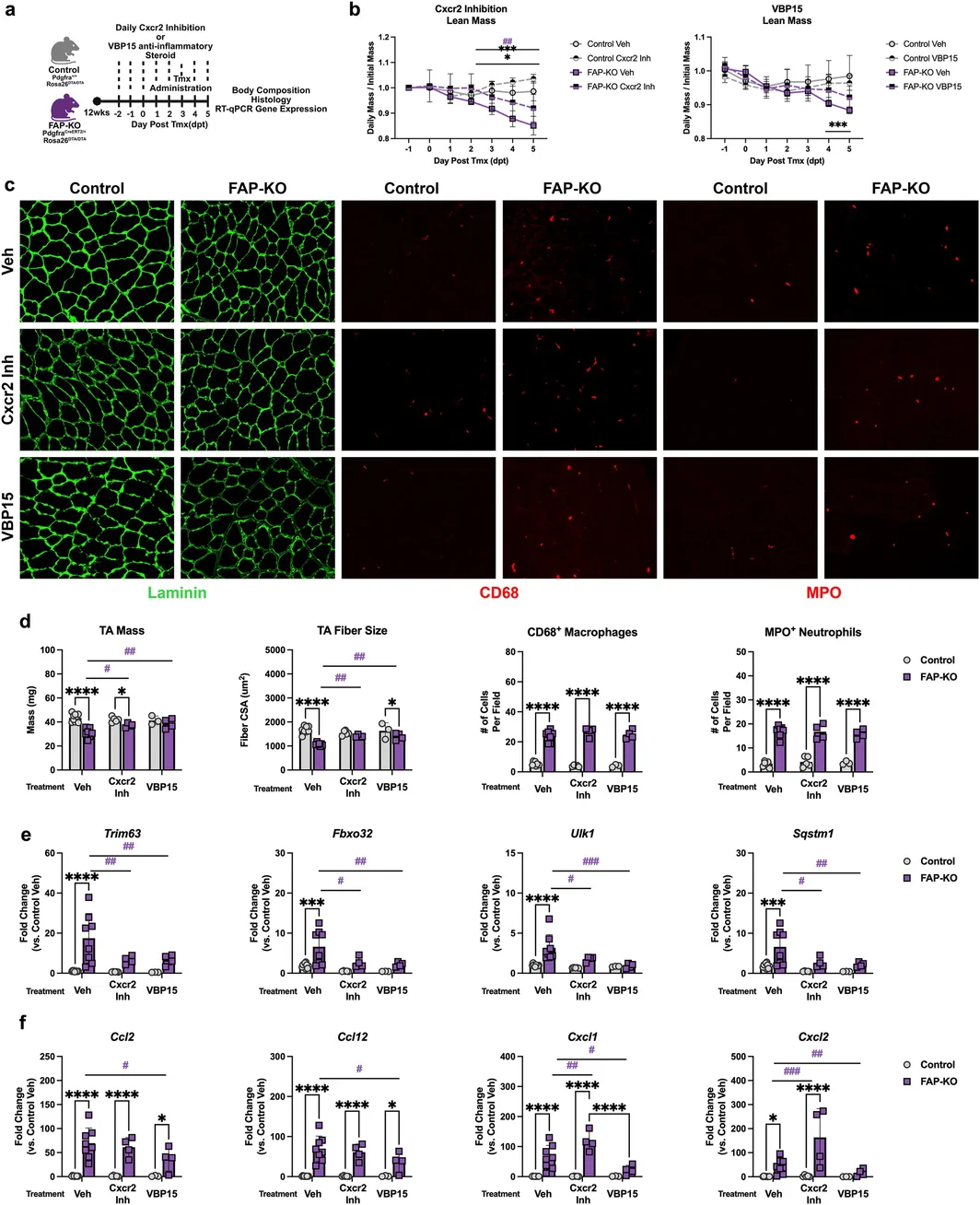
Pharmacological targeting of NF‐κB and the Cxcr2 axis partially attenuates FAP‐deletion‐induced muscle wasting and atrophic signalling. (a) Experimental schematic detailing the treatment timeline for the Cxcr2 antagonist (Cxcr2 Inh) or VBP15 administered alongside tamoxifen (Tmx)‐induced FAP clearance, with downstream tracking of body composition, histology, and transcriptomic profiles. (b) Longitudinal tracking of normalized daily lean mass under Cxcr2 inhibition (left) or VBP15 treatment (right), demonstrating a robust therapeutic rescue and prevention of rapid lean mass wasting in treated FAP‐KO cohorts. *N* = 4 ~ 6 per treatment per genotype. Cxcr2 inhibition: Two‐way ANOVA: genotype *p* < 0.0001, treatment *p* < 0.0001. VBP15: Two‐way ANOVA: genotype *p* = 0.0048, treatment *p* < 0.0001. (c) Representative immunofluorescence muscle cross‐sections for vehicle (Veh), Cxcr2 inhibitor (Cxcr2 Inh), and VBP15 treatment, stained for Laminin (green), CD68 (red, macrophage marker), and MPO (red, neutrophil marker) at 5 dpt. Scale bar = 100 um. (d) Quantitative metrics assessing absolute tibialis anterior (TA) muscle mass, mean myofiber cross‐sectional area (fibre CSA) and the spatial accumulation of interstitial CD68+ macrophages and MPO+ neutrophils at 5 dpt, confirming the maintenance of muscle mass and fibre size despite the unabated physical infiltration of both myeloid cell subsets. *N* = 4 ~ 6 per treatment per genotype. TA Mass: Two‐way ANOVA: genotype *p* < 0.0001, treatment *p* = 0.0868. TA fibre size: Two‐way ANOVA: genotype *p* < 0.0001, treatment *p* = 0.0850. CD68: Two‐way ANOVA: genotype *p* < 0.0001, treatment *p* = 0.7295. MPO: Two‐way ANOVA: genotype *p* < 0.0001, treatment *p* = 0.8102. (e) Relative mRNA expression analysis (fold change vs. Control Veh) of primary muscle‐specific E3 ubiquitin ligases (*Trim63*, *Fbxo32*) and key autophagy markers (*Ulk1*, *Sqstm1*) at 5 dpt, illustrating significant transcriptional suppression of these proteolytic pathways by both pharmacological interventions. *N* = 4 ~ 8 per treatment per genotype. *Trim63*: Two‐way ANOVA: genotype *p* = 0.0004, treatment *p* = 0.0479. *Fbxo32*: Two‐way ANOVA: genotype *p* = 0.0018, treatment *p* = 0.0044. *Ulk1*: Two‐way ANOVA: genotype *p* = 0.0013, treatment *p* = 0.0044. *Sqstm1*: Two‐way ANOVA: genotype *p* = 0.0018, treatment *p* = 0.0044. f: Relative mRNA expression profiling of local CC‐chemokines (*Ccl2*, *Ccl12*) and CXC‐chemokines (*Cxcl1*, *Cxcl2*) at 5 dpt, highlighting a distinct compensatory transcriptional feedback loop driving hyper‐elevated *Cxcl2* expression specifically in the Cxcr2 inhibitor‐treated FAP‐KO group. *N* = 4 ~ 8 per treatment per genotype. *Ccl2*: Two‐way ANOVA: genotype *p* < 0.0001, treatment *p* = 0.1791. *Ccl12*: Two‐way ANOVA: genotype *p* < 0.0001, treatment *p* = 0.1791. *Cxcl1*: Two‐way ANOVA: genotype *p* < 0.0001, treatment *p* = 0.0013. *Cxcl2*: Two‐way ANOVA: genotype *p* < 0.0001, treatment *p* = 0.002. Data are expressed as mean ± SD. Statistical significance was evaluated using a two‐way ANOVA followed by Tukey's post hoc test for multiple comparisons. Asterisks (*) indicate significant differences between genotypes under the same treatment condition (*p < 0.05, ***p < 0.001, ****p < 0.0001). Pound signs (#) indicate significant differences between therapeutic treatment channels or groups (#*p* < 0.05, ##*p* < 0.01, ###*p* < 0.001).

This systemic protection was corroborated by terminal anatomical and histological metrics. Both VBP15 and SB225002 administration significantly preserved absolute muscle wet weight and myofiber CSA compared to the FAP‐KO vehicle group at 5 dpt, although minor deficits persisted, with muscle mass marginally lower in the SB225002 cohort and myofiber CSA slightly depressed in the VBP15 cohort relative to controls (Figure 7c,d). Importantly, immunofluorescence analysis confirmed that neither pharmacological intervention altered the immune cell numbers infiltrating the interstitial space (Figure 7c,d). This confirms that mitigating muscle wasting requires targeted receptor‐level or transcriptional modulation to reduce inflammation rather than physical immune cell clearance.

At the molecular level, both Cxcr2 inhibition and VBP15 administration significantly reduced the transcription of muscle atrogenes at 5 dpt, although it was not fully corrected (Figure 7e). Transcriptional profiling of local chemokines revealed distinct mechanisms of action between the two therapeutic strategies. VBP15 broadly downregulated the expression of *Ccl2*, *Ccl12*, *Cxcl1* and *Cxcl2*, with the most pronounced suppression observed in the CXC‐ligands at 5 dpt (Figure 7f). Conversely, selective Cxcr2 receptor blockade provoked a marked compensatory upregulation of *Cxcl1* and *Cxcl2* expression (Figure 7f), demonstrating a classic feedback loop triggered by ligand‐receptor uncoupling during acute tissue stress. Collectively, these data demonstrate that targeted short‐term anti‐inflammatory interventions mitigate acute FAP deletion‐induced atrophy and establish that this protection is mediated, at least in part, via the localized Cxcl1/2‐Cxcr2 signalling axis.

## 4 Discussion

FAPs are increasingly recognized as essential regulators of skeletal muscle homeostasis, as their removal consistently induces profound muscle atrophy across multiple transgenic models [15, 16, 18]. Using an inducible FAP‐KO model, we confirmed that FAP deletion rapidly induces muscle atrophy in both male and female mice, independent of food and water consumption. Despite substantial reductions in muscle size and force production, we found no evidence of structural or functional NMJ impairment, nor secondary hallmarks of denervation, indicating that rapid atrophy after FAP deletion occurs independently of NMJ dysfunction. Guided by the observation that muscle loss developed concurrently with FAP ablation, we investigated the earliest cellular events following deletion, tying the atrophy to FAP cell death. FAP deletion triggered a robust inflammatory response characterized by marked immune cell infiltration and induction of chemokine expression, which preceded the atrogene upregulation. Pharmacological inhibition of CXCR2 inflammatory signalling attenuated muscle atrophy, whereas immune cell depletion failed to provide protection. These findings identify inflammation as a critical mediator of FAP deletion‐induced muscle wasting and implicate the CXCL1/2–CXCR2 axis as an important contributor to the inflammatory response. Ultimately, FAPs, themselves, become the culprit of triggering the inflammatory response through their death and may compound the extent of atrophy through the absence of their normal interactions with inflammatory cells. The early stages of transgenic induction may set the stage for subsequent requirements for FAPs in maintainence of muscle mass, but we assert that the early atrophy is not evidence for proactive protection of mass by FAPs.

Several mechanisms have been proposed to explain the muscle atrophy that follows FAP deletion. Early studies using fibroblast activation protein‐targeted ablation models identified a reduction in follistatin (Fst) levels following FAP loss, suggesting that FAPs serve as an important source of Fst within homeostatic skeletal muscle [18]. However, Fst decline occurred substantially later than *Trim63* and *Fbxo32* induction, indicating that reduced Fst signalling is an unlikely initiator of acute atrophy. More recently, FAP deletion‐induced muscle wasting has been attributed to NMJ degeneration through disruption of FAP‐derived BMP3B signalling, linking FAP dysfunction to age‐associated sarcopenia and NMJ instability [16, 34], counter to our findings. Consistent with previous reports, we observed that FAPs are enriched around the NMJ, supporting the existence of local interactions between FAPs and other synaptic cell populations. However, acute FAP ablation did not alter any aspect of NMJ patency. The discrepancy between our findings and previous reports may reflect important differences in experimental design, such as age of mice, where our experiments utilized older mice (> 12 weeks old). Indeed, age has been recognized as a major determinant of the contribution of satellite cells to muscle hypertrophy [11, 12]. It is therefore possible that the contribution of FAPs to NMJ maintenance is age‐dependent, with younger muscles displaying greater sensitivity to FAP loss than mature adult tissue. Despite a nonreciprocal relationship with the NMJ, FAP dynamics are actively modulated by alterations in neuromuscular integrity and Schwann cell function [35]. Therefore, the importance of investigating FAP behaviour remains, although the direction of interaction needs to be more carefully interpreted.

Our temporal analyses revealed that FAP deletion‐induced muscle wasting is preceded by a rapid and robust inflammatory response. Marked induction of inflammatory chemokines was detected as early as 3 dpt, preceding atrogene activation at 5 dpt. These findings led to one of the central conclusions of this study: infiltrating innate immune cells play a complex and context‐dependent role during FAP deletion‐induced muscle wasting. Inflammation has long been recognized as a major contributor to skeletal muscle wasting, particularly in cancer cachexia and other chronic diseases [36]. Consequently, strategies aimed at suppressing inflammatory cell populations have been explored as potential therapeutic approaches. For example, macrophage depletion has been reported to improve muscle phenotypes in cachectic settings [37], while neutrophil depletion can enhance regeneration by limiting excessive inflammation [38]. In contrast, our data demonstrate that both macrophages and neutrophils exert protective functions following acute FAP deletion. Depletion of either immune cell population exacerbated muscle loss and further increased atrogene expression, while macrophage depletion alone was sufficient to induce muscle atrophy in control mice. These phenomena could be explained by the ability of infiltrating macrophages to provide compensatory trophic support during this acute phase. However, despite substantial immune cell infiltration following FAP deletion, the expression of potent growth factors, including *Igf1*, *Fst*, *Hgf* and *Fgf2*, remained unchanged or was reduced. Although these whole‐muscle measurements cannot exclude cell‐specific changes in growth factor production, they do not support enhanced trophic factor production as the primary mechanism by which infiltrating immune cells protect against muscle atrophy. Rather, the protective effects of these immune populations likely involve mechanisms independent of direct growth factor supplementation. This supports that innate immune cells contribute to basal muscle homeostasis and may serve important functions in debris clearance and stress resolution following widespread progenitor cell death.

Pharmacological inhibition of inflammatory signalling produced effects opposite to those observed following immune cell depletion. Both the NF‐κB inhibitor Vamorolone (VBP15) and the CXCR2 antagonist SB225002 attenuated muscle wasting without altering immune cell infiltration. This indicates that inflammatory signalling, rather than immune cell abundance itself, is a primary driver of the atrophic response. This interpretation is consistent with previous studies demonstrating beneficial effects of NF‐κB inhibition in inflammatory muscle wasting conditions [37] and experimental evidence showing that activation of CXCR2 signalling is sufficient to induce muscle atrophy [39]. Together, our data support a model in which infiltrating myeloid cells provide a protective homeostatic function, whereas excessive NF‐κB‐dependent inflammation and CXCL1/2–CXCR2 signalling promote muscle wasting following FAP deletion.

A critical question that was not fully addressed in this study is the cellular source of the marked CXCL1 and CXCL2 induction. Based on previous reports and our findings, myofibers represent a plausible source of these chemokines during inflammatory stress [37], although contributions from other stromal or immune populations cannot be excluded. Future studies employing cell‐specific transcriptomic approaches will be necessary to identify the dominant source of CXCL1/2 signalling following FAP deletion. Nevertheless, the ability of both CXCR2 inhibition and NF‐κB blockade to attenuate muscle wasting strongly supports a causal role for inflammatory signalling in the development of the acute atrophic phenotype. Overall, these findings reveal that acute FAP deletion initiates an inflammatory program that drives muscle wasting through, at least in part, the CXCL1/2–CXCR2 signalling axis.

While the present study demonstrates that acute muscle atrophy following FAP deletion is, in part, inflammation‐mediated, our experimental design cannot fully distinguish between the effects of FAP loss and the consequences of their DTA‐induced cell death. Because mesenchymal stromal cells are broadly distributed throughout tissues, systemic ablation may trigger the release of damage‐associated molecular patterns (DAMPs) and other inflammatory mediators that could independently contribute to muscle wasting [40]. Consistent with this possibility, immune cell depletion produced mild atrophy in control mice and did not attenuate muscle loss in FAP‐deleted mice, suggesting that cellular death itself may adversely affect muscle homeostasis. Further, the liver and fat depots also displayed reduced masses, consistent with the presence of this cell population in these tissues. A complete separation of these mechanisms is inherently challenging, as selective removal of FAPs necessarily involves cell death. However, cell apoptosis was apparent as early as 1 dpt, while reduction of lean mass and fat mass were not apparent until 5 dpt; hence, the prolonged period between cell apoptosis and atrophy supports atrophy could be DAMPs independent and inflammation dependent. Indeed, the incomplete rescue achieved by anti‐inflammatory interventions suggests that inflammation alone does not account for the entire phenotype. These findings raise the possibility that the loss of FAP‐derived trophic support partially contributes to the early atrophic response. Among the growth factors examined, only *Igf1* and *Mstn* were significantly altered during the period of acute muscle loss. Although increased *Mstn* expression could potentially contribute to the atrophic response, the magnitude of this increase was modest. Similarly, despite the reduction in muscle *Igf1* following FAP depletion, our recent work demonstrated that selective deletion of *Igf1* from FAPs does not alter muscle mass under homeostatic conditions [19]. Together, these findings suggest that FAP‐derived trophic factors such as IGF‐I, although present in uninjured skeletal muscle, may not be essential for the maintenance of basal muscle mass in the absence of external stress. Furthermore, the recovery of total muscle *Igf1* expression to baseline levels following acute FAP ablation suggests that other cellular sources, such as immune cells, can compensate for the loss of FAP‐derived IGF‐I and contribute to maintenance of the muscle IGF‐I pool. Nonetheless, other FAP‐derived factors are likely involved in maintaining muscle homeostasis. Supporting this concept, transplantation of FAPs into homeostatic muscle partially rescues muscle atrophy following FAP deletion [15]. In addition to providing trophic support, FAPs may regulate local immune homeostasis. Previous studies have shown bidirectional interactions between FAPs and immune cells [19], where FAPs can exert immunomodulatory functions under basal conditions. Collectively, these findings highlight the importance of interpreting FAP ablation studies with caution and underscore the need for future investigations aimed at defining the specific mechanisms through which FAPs maintain skeletal muscle homeostasis.

## Ethics Statement

All authors have certified that they comply with the Ethical guidelines for authorship and publishing in the *Journal of Cachexia, Sarcopenia and Muscle*.

## Conflicts of Interest

The authors declare no conflicts of interest.

## Supporting information


**Figure S1:** Dynamics of fat mass during Tmx‐induced FAP deletion and interventions. (a) Longitudinal fat mass tracking normalized to initial fat mass during Tmx administration without additional rescue treatment demonstrates apparent fat loss occurs at 5 dpt. *N* = 25 ~ 28 per genotype. Two‐way ANOVA: genotype *p* = 0.0313, timepoint *p* < 0.0001. (b) Longitudinal fat mass tracking normalized to initial fat mass during Tmx administration with double macrophage and neutrophil depletion. *N* = 4 ~ 6 per timepoint per genotype per treatment. Two‐way ANOVA: genotype *p* = 0.3632, timepoint *p* = 0.9285. (c) Longitudinal fat mass tracking normalized to initial fat mass during Tmx administration with macrophage depletion. *N* = 4 ~ 6 per timepoint per genotype per treatment. Two‐way ANOVA: genotype *p* = 0.0030, timepoint *p* < 0.0001. (d) Longitudinal fat mass tracking normalized to initial fat mass during Tmx administration with neutrophil depletion. *N* = 4 ~ 6 per timepoint per genotype per treatment. Two‐way ANOVA: genotype *p* < 0.0001, timepoint *p* = 0.0987. (e) Longitudinal fat mass tracking normalized to initial fat mass during Tmx administration with Cxcr2 inhibition. *N* = 4 ~ 6 per timepoint per genotype per treatment. Two‐way ANOVA: genotype *p* < 0.0001, timepoint *p* = 0.0038. (f) Longitudinal fat mass tracking normalized to initial fat mass during Tmx administration with VBP15 treatment. *N* = 4 ~ 6 per timepoint per genotype per treatment. Two‐way ANOVA: genotype *p* < 0.0001, timepoint *p* = 0.0276. Data are expressed as mean ± SD. Statistical significance was evaluated using a two‐way ANOVA followed by Tukey's post hoc test for multiple comparisons. Asterisks (*) indicate significant differences between genotypes under the same deletion condition (* *p* < 0.05; ** *p* < 0.01; *** *p* < 0.001; **** *p* < 0.0001). Pound signs (#) indicate significant differences between deletion methods or experimental treatment groups (# *p* < 0.05; ## *p* < 0.01; ### *p* < 0.001; #### *p* < 0.0001).
**Figure S2:** FAP deletion‐induced lean mass loss is independent of food and water intake. (a) Longitudinal food intake tracking demonstrates that Tmx treatment reduces food intake in Control and FAP‐KO mice at 1–2 dpt, but FAP deletion does not affect food intake. N = 4 ~ 13 per timepoint and genotype. Two‐way ANOVA: genotype p = 0.1417, timepoint p < 0.0001. (b) Longitudinal water intake tracking demonstrates Tmx treatment results in increased water intake in Control and FAP‐KO mice at 4–5 dpt, but FAP deletion does not affect water intake. N = 4 ~ 13 per timepoint per genotype per genotype. Two‐way ANOVA: genotype p = 0.0089, timepoint p = 0.0004. Data are expressed as mean ± SD. Statistical significance was evaluated using a two‐way ANOVA followed by Tukey's post hoc test for multiple comparisons. Asterisks (*) indicate significant differences between genotypes under the same deletion condition and timepoint (** p < 0.01). Black is control oil vs. control Tmx. Pound signs (#) indicate significant differences within genotype and between timepoints (# p < 0.05; ## p < 0.01). Purple is FAP‐KO Tmx, and black is control Tmx.
**Figure S3:** Tmx induced diptheria Toxin (dTA) expression drives early apoptosis in FAPs. (a) Representative immunofluorescence images of muscle sections detecting PDGFRα (Red), Cleaved‐Caspase3 (Green) and Nuclei (DAPI). Scale bar = 50 μm. (b) Quantification of the number of apoptotic FAPs during Tmx administration revealed apparent FAP apoptosis at 1 dpt. N = 3 per timepoint and genotype. Two‐way ANOVA: genotype p < 0.0001, timepoint p < 0.0001. Data are expressed as mean ± SD. Statistical significance was evaluated using a two‐way ANOVA followed by Tukey's post hoc test for multiple comparisons. Asterisks (*) indicate significant differences between genotypes at the same timepoint (**** p < 0.0001). Pound signs (#) indicate significant differences between timepoints within the same genotype (#### p < 0.0001).
**Figure S4:** FAP deletion alters whole tissue growth factor expression and modestly increases circulating IL‐6. (a) FAP deletion results in a transient reduction of Igf1 expression in muscle at 5 dpt. N = 3 ~ 8 per timepoint per genotype. Two‐way ANOVA: genotype p = 0.0019, timepoint p = 0.62. (b) FAP deletion results in lower Fst transcription in muscle at 28 dpt. N = 3 ~ 8 per timepoint per genotype. Two‐way ANOVA: genotype p = 0.1109, timepoint p = 0.2186. (c) FAP deletion does not alter Hgf transcript levels in muscle. N = 3 ~ 8 per timepoint per genotype. Two‐way ANOVA: genotype p = 0.807, timepoint p = 0.5956. (d) FAP deletion does not alter Fgf2 transcript levels in muscle. N = 3 ~ 8 per timepoint per genotype. Two‐way ANOVA: genotype p = 0.8291, timepoint p = 0.3602. (e) FAP deletion results in transiently increased Mstn expression in muscle at 1‐5 dpt. N = 3 ~ 8 per timepoint per genotype. Two‐way ANOVA: genotype p = 0.0331, timepoint p < 0.0001. (f) FAP deletion results in mild but significant transient elevation of circulating IL‐6 levels. N = 3 ~ 6 per timepoint and genotype. Two‐way ANOVA: genotype p = 0.0008, timepoint p = 0.0335. Data are expressed as mean ± SD. Statistical significance was evaluated using a two‐way ANOVA followed by Tukey's post hoc test for multiple comparisons. Asterisks (*) indicate significant differences between genotypes under the same timepoint (* p < 0.05; ** p < 0.01; *** p < 0.001; **** p < 0.0001). Pound signs (#) indicate significant differences within the same genotype between different timepoints (# p < 0.05; ## p < 0.01; ### p < 0.001).
**Table S1:** Tissue masses across time points (Pre‐Tmx, 1, 3, 5, and 28 dpt).

## Data Availability

The data that support the findings of this study are available from the corresponding author upon reasonable request.
