# Supplementary material for: Fibro‐Adipogenic Progenitor Ablation Triggers Muscle Atrophy Through Cell Death‐Induced Inflammation
Source: J Cachexia Sarcopenia Muscle. 2026 Sep 29;17(5):e70393. doi: 10.1002/jcsm.70393 (PMC13624542; doi:10.1002/jcsm.70393)
Supplement: Supplementary file 1 [file JCSM-17-e70393-s001.docx]

**Supplemental Materials**

**Figure S1: Dynamics of fat mass during Tmx induced FAP Deletion and interventions.**

a: Longitudinal fat mass tracking normalized to initial fat mass during Tmx administration without additional rescue treatment demonstrates apparent fat loss occurs at 5dpt. N=25~28 per genotype. Two-way ANOVA: genotype p=0.0313, timepoint p<0.0001.

b: Longitudinal fat mass tracking normalized to initial fat mass during Tmx administration with double macrophage and neutrophil depletion. N=4~6 per timepoint per genotype per treatment. Two-way ANOVA: genotype p=0.3632, timepoint p=0.9285.

c: Longitudinal fat mass tracking normalized to initial fat mass during Tmx administration with macrophage depletion. N=4~6 per timepoint per genotype per treatment. Two-way ANOVA: genotype p=0.0030, timepoint p<0.0001.

d: Longitudinal fat mass tracking normalized to initial fat mass during Tmx administration with neutrophil depletion. N=4~6 per timepoint per genotype per treatment. Two-way ANOVA: genotype p<0.0001, timepoint p=0.0987.

e: Longitudinal fat mass tracking normalized to initial fat mass during Tmx administration with Cxcr2 inhibition. N=4~6 per timepoint per genotype per treatment. Two-way ANOVA: genotype p<0.0001, timepoint p=0.0038.

f: Longitudinal fat mass tracking normalized to initial fat mass during Tmx administration with VBP15 treatment. N=4~6 per timepoint per genotype per treatment. Two-way ANOVA: genotype p<0.0001, timepoint p=0.0276.

Data are expressed as mean ± SD. Statistical significance was evaluated using a two-way ANOVA followed by Tukey's post-hoc test for multiple comparisons. Asterisks (*) indicate significant differences between genotypes under the same deletion condition (* P < 0.05; ** P < 0.01; *** P < 0.001; **** P < 0.0001). Pound signs (#) indicate significant differences between deletion methods or experimental treatment groups (# P < 0.05; ## P < 0.01; ### P < 0.001; #### P < 0.0001).

**Figure S2. FAP deletion-induced lean mass loss is independent of food and water intake.**

a: Longitudinal food intake tracking demonstrates that Tmx treatment reduces food intake in Control and FAP-KO mice at 1-2 dpt, but FAP deletion does not affect food intake. N=4~13 per timepoint and genotype. Two-way ANOVA: genotype p=0.1417, timepoint p<0.0001.

b: Longitudinal water intake tracking demonstrates Tmx treatment results in increased water intake in Control and FAP-KO mice at 4-5 dpt, but FAP deletion does not affect water intake. N=4~13 per timepoint per genotype per genotype. Two-way ANOVA: genotype p=0.0089, timepoint p=0.0004.

Data are expressed as mean ± SD. Statistical significance was evaluated using a two-way ANOVA followed by Tukey's post-hoc test for multiple comparisons. Asterisks (*) indicate significant differences between genotypes under the same deletion condition and timepoint (** P < 0.01). Black is Control Oil vs Control Tmx. Pound signs (#) indicate significant differences within genotype and between timepoints (# P < 0.05; ## P < 0.01). Purple is FAP-KO Tmx, and black is Control Tmx.

**Figure S3: Tmx induced diptheria Toxin (dTA) expression drives early apoptosis in FAPs.**

a: Representative immunofluorescence images of muscle sections detecting PDGFRα (Red), Cleaved-Caspase3 (Green), and Nuclei (DAPI). Scale bar = 50µm.

b: Quantification of the number of apoptotic FAPs during Tmx administration revealed apparent FAP apoptosis at 1dpt. N=3 per timepoint and genotype. Two-way ANOVA: genotype p<0.0001, timepoint p<0.0001.

Data are expressed as mean ± SD. Statistical significance was evaluated using a two-way ANOVA followed by Tukey's post-hoc test for multiple comparisons. Asterisks (*) indicate significant differences between genotypes at the same timepoint (**** P < 0.0001). Pound signs (#) indicate significant differences between timepoints within the same genotype (#### P < 0.0001).

**Figure S4. FAP deletion alters whole tissue growth factor expression and modestly increases circulating IL-6.**

a: FAP deletion results in a transient reduction of *Igf1* expression in muscle at 5dpt. N=3~8 per timepoint per genotype. Two-way ANOVA: genotype p=0.0019, timepoint p=0.62.

b: FAP deletion results in lower *Fst* transcription in muscle at 28dpt. N=3~8 per timepoint per genotype. Two-way ANOVA: genotype p=0.1109, timepoint p=0.2186.

c: FAP deletion does not alter *Hgf* transcript levels in muscle. N=3~8 per timepoint per genotype. Two-way ANOVA: genotype p=0.807, timepoint p=0.5956

d: FAP deletion does not alter *Fgf2* transcript levels in muscle. N=3~8 per timepoint per genotype. Two-way ANOVA: genotype p=0.8291, timepoint p=0.3602.

e: FAP deletion results in transiently increased *Mstn* expression in muscle at 1-5dpt. N=3~8 per timepoint per genotype. Two-way ANOVA: genotype p=0.0331, timepoint p<0.0001.

f: FAP deletion results in mild but significant transient elevation of circulating IL-6 levels. N=3~6 per timepoint and genotype. Two-way ANOVA: genotype p=0.0008, timepoint p=0.0335.

Data are expressed as mean ± SD. Statistical significance was evaluated using a two-way ANOVA followed by Tukey's post-hoc test for multiple comparisons. Asterisks (*) indicate significant differences between genotypes under the same timepoint (* P < 0.05; ** P < 0.01; *** P < 0.001; **** P < 0.0001). Pound signs (#) indicate significant differences within the same genotype between different timepoints (# P < 0.05; ## P < 0.01; ### P < 0.001).

**Table S1. Tissue masses across time points (Pre-Tmx, 1, 3, 5, and 28 dpt)**

| **Timepoint** | **Tissues** | **Genotype** | | | |  |
| --- | --- | --- | --- | --- | --- | --- |
|  |  | **Control+Tmx (mg) (n=3~5)** | | **FAP-KO+Tmx (mg) (n=3~5)** | | **Significance** |
| Pre-Tmx | TA | 41.0±3.3 | | 44.1±3.9 | | p=0.352, ns |
|  | Liver | 1299.8±242.5 | | 1418.6±125.0 | | p=0.494, ns |
|  | Heart | 131.55±18.57 | | 133.2±5.6 | | p= 0.891, ns |
|  | Spleen | 81.9±17.1 | | 76.7±19.2 | | p=0.745, ns |
|  | Adipose Depot | 18.5±4.9 | | 21.8±3.7 | | p=0.405, ns |
|  | Kidney | 208.4±34.4 | | 199.0±35.9 | | p=0.763, ns |
| 1 dpt | TA | 38.3±3.5 | | 36.6±2.2 | | p=0.500, ns |
|  | Liver | 910.6±102.9 | | 779.5±169.0 | | p=0.315, ns |
|  | Heart | 116.6±14.1 | | 92.3±3.5 | | p=0.073, ns |
|  | Spleen | 78.8±20.0 | | 80.6±33.9 | | p=0.940, ns |
|  | Adipose Depot | 27.7±11.5 | | 22.3±5.2 | | p=0.506, ns |
|  | Kidney | 136.4±8.2 | | 133.9±8.6 | | p=0.735, ns |
| 3 dpt | TA | 47.6±7.0 | | 39.7±0.8 | | p=0.160, ns |
|  | Liver | 1053.0±161.8 | | 886.1±136.0 | | p=0.243, ns |
|  | Heart | 110.7±16.12 | | 102.4±6.7 | | p=0.456, ns |
|  | Spleen | 71.63±8.63 | | 92.4±0.7* | | **p=0.014, *** |
|  | Adipose Depot | 28.1±6.9 | | 27.8±2.7 | | p=0.941, ns |
|  | Kidney | 142.0±22.9 | | 162.1±35.7 | | p=0.456, ns |
| 5 dpt | TA | 54.8±4.3 | | 42.0±2.7** | | **p=0.004, **** |
|  | Liver | 1371.0±184.2 | | 1062.0±156.7 | | p=0.060, ns |
|  | Heart | 139.4±29.6 | | 124.6±19.3 | | p=0.450, ns |
|  | Spleen | 100.0±15.3 | | 72.3±11.6* | | **p=0.035, *** |
|  | Adipose Depot | 39.1±4.7 | | 36.4±11.8 | | p=0.752, ns |
|  | Kidney | 142.0±22.9 | | 162.2±35.7 | | p=0.773, ns |
| 28 dpt |  | **Control** | | **FAP-KO** | **FAP-KO** |  |
|  |  | **Oil** | **Tmx** | **Oil** | **Tmx** |  |
|  | TA | 45.6±5.7 | 49.5±5.8 | 49.2±8.4 | 33.2±5.3 ***, ### | Tmx Control vs. Tmx FAP-KO p=0.0004, ***  Oil FAP-KO vs. Tmx FAP-KO p=0.0004, *** |
|  | EDL | 12.5±1.9 | 10.9±1.6 | 12.3±1.5 | 5.4±0.7 ****, #### | Tmx Control vs Tmx FAP-KO p<0.0001, ****  Oil FAP-KO vs. Tmx FAP-KO p<0.0001, **** |
|  | SOL | 13.1±1.9 | 11.6±1.3 | 13.8±2.6 | 6.7±0.8 ****, #### | Tmx Control vs Tmx FAP-KO p<0.0001, ****  Oil FAP-KO vs. Tmx FAP-KO p<0.0001, **** |
|  | Liver | 1158.4±208.4 | 1156.4±207.9 | 1197.1±170.8 | 964.1±205.5 *, # | Tmx Control vs Tmx FAP-KO p=0.024, *  Oil FAP-KO vs. Tmx FAP-KO p=0.034, * |
|  | Heart | 113.0±16.2 | 126.5±25.4 | 110.8±16.6 | 102.9±15.1 |  |
|  | Spleen | 107.3±17.6 | 98.9±20.6 | 81.1±9.1 | 57.6±16.5 ***, ### | Tmx Control vs Tmx FAP-KO p=0.0003, ***  Oil FAP-KO vs. Tmx FAP-KO p=0.0005, *** |
|  | Adipose Depot | 21.8±4.8 | 17.3±4.9 | 22.2±7.6 | 13.4±4.6 # | Oil FAP-KO vs. Tmx FAP-KO p=0.035, * |
|  | Kidney | 177.6±39.2 | 166.3±36.4 | 168.7±25.2 | 155.7±26.4 |  |

For Pre-Tmx, 1, 3, and 5 dpt, statistical analysis was performed using two-tailed Student t-test. For 28 dpt, statistical analysis was performed using Two-way ANOVA followed by Tukey’s post-hoc test for multiple comparisons. Asterisks (*) indicate significant differences between genotypes with the same treatment (* P < 0.05; *** P < 0.001; **** P < 0.0001). Pound signs (#) indicate significant differences between treatments within the same genotype (# P < 0.05; ### P < 0.001; #### P < 0.0001).

**Additional Methods**

*Ex-vivo* muscle contraction

Isolated EDL and SOL muscles from ketamine/xylazine-anesthetized mice were secured with silk sutures at the myotendinous junctions and mounted in an ex-vivo organ bath (Aurora Scientific) containing oxygenated (95% O2, 5% CO2) Ringer’s solution (119 mM NaCl, 4.7 mM KCl, 2.5 mM CaCl_2_, 1.2 mM KH_2_PO_4_, 1.2 mM MgSO_4_, 25 mM NaHCO_3_, 11 mM glucose) at 25°C. Tissues were attached to a dual-mode force transducer (Model 300C; Aurora Scientific) and stimulated via parallel platinum electrodes. Optimal length (Lo) was established using single twitch stimulations (0.2 ms pulse width). Force-frequency relationships were assessed using 300 ms (EDL) or 500 ms (SOL) stimulus trains at 10–150 Hz (EDL) or 10–120 Hz (SOL) with 2-minute rest intervals; peak absolute isometric force (Po) was captured at supramaximal frequency. Structural vulnerability was evaluated in EDL muscles via 5 eccentric contraction cycles (3-minute intervals): at 150 ms into a 120 Hz isometric contraction, a 10% Lo stretch was executed at 2.0 Lo/s, held for the remainder of the train, and returned to Lo. Force deficits were normalized to the first cycle. Specific force (N/cm²) was determined by dividing Po by the physiological cross-sectional area: pCSA = Muscle Mass (mg) / [Lo (mm) x (Lf/Lo) x Density], where mammalian muscle density is 1.06 mg/mm³, and the fiber-to-muscle length ratio (Lf/Lo) is 0.45 for EDL and 0.71 for SOL. Data were analyzed using ASI Commercial Software.

Direct and Nerve Mediated Muscle Contraction

Muscles were subjected to isolated mechanical measurements as described above, with modification to accommodate muscle contraction via stimulation of sciatic nerve that carry motor axons innervating the soleus muscle. Briefly, soleus muscles were dissected with their innervation intact to the sciatic notch [49] and pinned to a Sylgard-coated dish containing a modiﬁed Ringer’s solution (119 mM NaCl, 4.74 mM KCl, 3.36 mM CaCl_2_, 1.18 mM KH_2_PO_4_, 1.18 mM MgSO_4_, 25 mM HEPES, and 2.75 mM glucose) gas-equilibrated with 95% O2 -5% CO2. Silk suture 6 – 0 was tied at the myotendinous junctions. The optimum length (Lo) of the muscle was found by performing a series of twitches, each with an incremental increase in the length of the muscle, until the length that yielded the maximum twitch force was found. Subsequently, each muscle preparation was subjected to (1) a maximum isometric force test and (2) a force-frequency test. For each of the tests, the soleus muscle was stimulated first through the nerve with its distal cut end placed within a suction electrode followed by direct stimulation of the muscle using platinum electrodes located on each side of the muscle belly. The isometric force tests were performed by stimulating the soleus at 100Hz for 500ms, with a 300 second rest period between stimulations. The force-frequency test was performed using 500 ms trains with stimulation frequencies (in Hz) at 5, 10, 25, 50, 75, 120 and 150 with a resting period of 180 seconds between stimulations.

Tissue Collection and Preparation

Following euthanasia, hindlimb muscles were harvested and either snap-frozen directly in liquid nitrogen for biochemical assays or embedded in pre-chilled Optimal Cutting Temperature (OCT) compound and frozen in liquid-nitrogen-cooled isopentane for histology. Inguinal adipose depots were dissected from the subcutaneous inguinal region adjacent to the proximal hindlimb. Other organs were collected using standard dissection procedures. Excised tissues were cleared of excess connective tissue and weighed to determine tissue wet mass. All tissues were stored at -80°C. For histology, muscles were equilibrated in a Leica Cryostat (S3050) at -25°C for 30 minutes, and 10 µm transverse sections were collected onto adhesive slides.

Immunofluorescence Staining

Cryosections were equilibrated at RT for 30 minutes, fixed with 4% PFA for 10 minutes, and washed three times in PBS containing 0.1% Tween-20 (PBST). Sections were blocked for 1 hour at RT in blocking buffer (5% BSA, 0.3% Triton-X 100, and 0.2% sodium azide in PBS) and incubated with primary antibodies overnight at 4°C. Sections were washed with PBST, incubated with fluorophore-conjugated secondary antibodies for 1 hour at RT, washed again, and mounted using Fluoromount-G.

RT-qPCR

Frozen tissues were pulverized, lysed in Trizol Reagent, and phase-separated using chloroform. Total RNA was purified using Qiagen RNeasy Mini columns with on-column DNase digestion. RNA concentration was quantified via NanoDrop, and 500–1000 ng was reverse-transcribed to cDNA. Quantitative PCR was performed on a QuantStudio-3 system using 10 ng of cDNA per well combined with primers pairs targeting genes of interest and SYBR Green Master Mix. Samples were run in duplicate and the results averaged. Fold changes in gene expression were calculated by ddCt with Hprt used as the reference housekeeping gene.

Serum Collection and IL-6 Measurements

Whole blood was collected by cardiac puncture from mice under isoflurane anesthesia. Blood was allowed to clot at room temperature for 30 min, followed by centrifugation at 5,000 × g for 15 min. The resulting serum was collected and used for measurements of circulating IL-6 levels using a mouse IL-6 ELISA kit (R&D Systems) according to the manufacturer’s instructions. This kit has a sensitivity of 1.8 pg/mL and <0.5% cross reactivity with related molecules. Serum samples were run in duplicate, with 50 µL loaded per well. IL-6 concentrations were calculated from the mouse IL-6 kit standard.

Food and Water Consumption

Food and water intake were monitored from 4 days before Tmx administration through 5 days post-Tmx. At the beginning of the monitoring period, food and water were provided at predetermined amounts and were not replenished throughout the monitoring period. Food and water were weighed each day between 8-9 am using a calibrated scale, and daily consumption was calculated from the change in weight between consecutive measurements. All procedures were performed in accordance with the UF IACUC approval.

**Abbreviation Table**

| **Full Name** | **Abbreviation** |
| --- | --- |
| Fibro-Adipogenic Progenitors | FAPs |
| Neuromuscular Junction | NMJ |
| Vamorolone | VBP15 |
| Satellite Cells | SCs |
| Insulin-like Growth Factor I | IGF-I |
| Follistatin | Fst |
| Platelet-derived growth factor receptor alpha | PDGFRα |
| Tibialis Anterior | TA |
| Extensor Digitorum Longus | EDL |
| Soleus | SOL |
| Bungarotoxin | BTX |
| Diphtheria Toxin Subunit A | DTA |
| Tamoxifen | Tmx |
| Myeloperoxidase | MPO |
| Day Post Tamoxifen | Dpt |
| Cross-Sectional Area | CSA |
| TNFa | Tumor Necrosis Factor Alpha |
| TGFb | Transforming Growth factor Beta |
| IL | Interleukin |
| Myostatin | Mstn |
| Hepatocyte Growth Factor | Hgf |
| Fibroblast Growth Factor 2 | Fgf2 |

**Primary Antibodies**

| **Antibody** | **Company** | **Catalog** | **Dilution** |
| --- | --- | --- | --- |
| Anti-PDGFRα | R&D | AF-1062 | 1:250 |
| Anti-CD68 | Biolegend | 137001 | 1:500 |
| Anti-Laminin | Invitrogen | PA1-16730 | 1:500 |
| Anti-MPO | R&D | AF-3667 | 1:250 |
| Anti-Ly6G(1A8) | Absolute Antibody | Ab00295-2.0 | N/A |
| Anti-Cleaved-Cas3 | Cell Signaling | 9661T | 1:250 |

**Secondary Antibodies**

| **Antibody** | **Company** | **Catalog** | **Dilution** |
| --- | --- | --- | --- |
| 488 Donkey Anti-Rabbit | Invitrogen | A-21206 | 1:500 |
| 568 Donkey Anti-Goat | Invitrogen | A-11057 | 1:500 |
| 568 Goat Anti-Rat | Invitrogen | A-11077 | 1:500 |
| DAPI | Invitrogen | D3571 | 1:1000 |

**Materials and Reagents**

| **Reagent** | **Company** | **Catalog #** |
| --- | --- | --- |
| G2 Master Mix Taq Pol | Promega | M7882 |
| Tamoxifen | Biosynth | FT27994-100g |
| Tissue-Tek O.C.T Compound | Sakura | 4583 |
| Paraformaldehyde 16% Aqueous Solution | Electron Microscopy Science | 15710 |
| Fluoromount-G^TM^ | Invitrogen | 00-4958-02 |
| Trizol Reagent | Thermo Fisher | 15596026 |
| RNeasy Mini Kit | Qiagen | 74104 |
| RNase-Free DNase Set | Qiagen | 79256 |
| High Capacity cDNA Reverse Transcription Kit | Thermo Fisher | 4368814 |
| 2x Universal SYBR Green Fast qPCR Mix | Abclonal | RK21203 |
| Clodrosome | Encapsula Nano Sciences | CLD-8909 |
| Encapsome | Encapsula Nano Sciences | CLD-8910 |
| SB 225002 (Cxcr2 Antagonist) | Selleckchem | S7651 |
| Vamorolone | MedChemExpress | HY-109017 |
| Mouse IL-6 Quantikine ELISA Kit | R&D Systems | M6000B-1 |

**Primer Sequences**

| **Gene name** | **Primer Direction** | **Sequence** |
| --- | --- | --- |
| *Pdgfra^Cre-ERT2^* Mut | Mut Fwd | ATC GCA TTC CTT GCA AAA GT |
| *Pdgfra^Cre-ERT2^* Com | Com Rev | AGG CCC ACA GAA CAT GGA C |
| *Rosa26^DTA^* | Mut Fwd | CGA CCT GCA GGT CCT CG |
| *Rosa26^DTA^* | Mut Rev | CTC GAG TTT GTC CAA TTA TGT CAC |
| *Rosa26^DTA^* | WT Fwd | CCA AAG TCG CTC TGA GTT GTT ATC |
| *Rosa26^DTA^* | WT Rev | GAG CGG GAG AAA TGG ATA TG |
| *Hprt* | Fwd | CATAACCTGGTTCATCATCGC |
| *Hprt* | Rev | TCCTCCTCAGACCGCTTTT |
| *Ccl2* | Fwd | GCAGAGAGCCAGACGGG |
| *Ccl2* | Rev | ACAGCTTCTTTGGGACACCT |
| *Ccl4* | Fwd | CAAACCTAACCCCGAGCAA |
| *Ccl4* | Rev | TTGGTCAGGAATACCACAGC |
| *Ccl5* | Fwd | TGCAGAGGACTCTGAGACAGC |
| *Ccl5* | Rev | GAGTGGTGTCCGAGCCATA |
| *Ccl12* | Fwd | TTAAAAACCTGGATCGGAACCAA |
| *Ccl12* | Rev | GCATTAGCTTCAGATTTACGGGT |
| *Ccl17* | Fwd | GCTGCTTTCAGCATCCAAGTG |
| *Ccl17* | Rev | CCAGGGACACCGACTACTG |
| *Ccr2* | Fwd | AGGAGCCATACCTGTAAATGC |
| *Ccr2* | Rev | TGTGGTGAATCCAATGCCCT |
| *Cxcl1* | Fwd | GGCTGGGATTCACCTCAAGAACATC |
| *Cxcl1* | Rev | AGTGTTGTCAGAAGCCAGCGTTCAC |
| *Cxcl2* | Fwd | AAAATCATCCAAAAGATACTGAACAA |
| *Cxcl2* | Rev | CTTTGGTTCTTCCGTTGAGG |
| *Cxcl5* | Fwd | ATTTCTGTTGCTGTTCACGC |
| *Cxcl5* | Rev | GTTTAGCTATGACTTCCACCGT |
| *Cxcl8* | Fwd | TCAATGCCTGAAGACCCTGCCAA |
| *Cxcl8* | Rev | TGGGTTCTTCCGTTGAGGGACAGC |
| *Cxcl10* | Fwd | CTAGCTCAGGCTCGTCAGTT |
| *Cxcl10* | Rev | CCCTTGGGAAGATGGTGGTT |
| *Il-1a* | Fwd | ACGGCTGAGTTTCAGTGAGACC |
| *Il-1a* | Rev | CACTCTGGTAGGTGTAAGGTGC |
| *Il-1b* | Fwd | CAACCAACAAGTGATATTCTCCAT |
| *Il-1b* | Rev | GGGTGTGCCGTCTTTCATTA |
| *Il-2* | Fwd | CCCAGGATGCTCACCTTCAA |
| *Il-2* | Rev | CCGCAGAGGTCCAAGTTCAT |
| *Il-4* | Fwd | CGAATGTACCAGGAGCCATATC |
| *Il-4* | Rev | TCTCTGTGGTGTTCTTCGTTG |
| *Il-6* | Fwd | GCTACCAAACTGGATATAATCAGGA |
| *Il-6* | Rev | CCAGGTAGCTATGGTACTCCAGAA |
| *Il-10* | Fwd | CAGAGCCACATGCTCCTAGA |
| *Il-10* | Rev | GTCCAGCTGGTCCTTTGTTT |
| *Tnfa* | Fwd | ATTCGAGTGACAAGCCTGTAG |
| *Tnfa* | Rev | AGATAGCAAATCGGCTGACG |
| *Ifng* | Fwd | CCTAGCTCTGAGACAATGAACG |
| *Ifng* | Rev | TTCCACATCTATGCCACTTGAG |
| *Csf1* | Fwd | GACAACACCCCCAATGCTAA |
| *Csf1* | Rev | GGTCACCACATCTCGGCTA |
| *Csf2* | Fwd | TGTCACGTTGAATGAAGAGGT |
| *Csf2* | Rev | GGCTGTCTATGAAATCCGCA |
| *Tgfb1* | Fwd | ATACGCCTGAGTGGCTGTCT |
| *Tgfb1* | Rev | CTGATCCCGTTGATTTCCA |
| *Ulk1* | Fwd | ACT TTG GAT TCG CTC GGT AC |
| *Ulk1* | Rev | GGT CAG CCT TTC CAT CGT AG |
| *Sqstam1* | Fwd | CCCAGTGTCTTGGCATTCTT |
| *Sqstam1* | Rev | AGGGAAAGCAGAGGAAGCTC |
| *Trim63* | Fwd | GGTGCCTACTTGCTCCTT |
| *Trim63* | Rev | ACC AGC ATG GAG ATG CAG TTA |
| *Fbxo32* | Fwd | GCAGAGAGTCGGCAAGTC |
| *Fbxo32* | Rev | CAGGTCGGTGATCGTGAG |
| *Hgf* | Fwd | GTCCTGAAGGCTCAGACTTGGT |
| *Hgf* | Rev | CCAGCCGTAAATACTGCAAGTGG |
| *Fgf2* | Fwd | AAGCGGCTCTACTGCAAGAACG |
| *Fgf2* | Rev | CCTTGATAGACACAACTCCTCTC |
| *Mstn* | Fwd | AACCTTCCCAGGACCAGGAGAA |
| *Mstn* | Rev | GGCTTCAAAATCGACCGTGAGG |
| *Igf1* | Fwd | GGACCGAGGGGCTTTTACTT |
| *Igf1* | Rev | GTGGGGCACAGTACATCTCC |
| *Fst* | Fwd | CTGTCTGATGAGAACGCTGACTG |
| *Fst* | Rev | AGACACAGCGATTGCAGTCCAC |

**Additional References**

41.	Madaro L, Passafaro M, Sala D, Etxaniz U, Lugarini F, Proietti D, et al. Denervation-activated STAT3-IL-6 signalling in fibro-adipogenic progenitors promotes myofibres atrophy and fibrosis. Nat Cell Biol. 2018;20:917-27. doi:10.1038/s41556-018-0151-y

42.	Clamon G, Byrne MM, Talbert EE. Inflammation as a Therapeutic Target in Cancer Cachexia. Cancers (Basel). 2022;14:doi:10.3390/cancers14215262

43.	Pryce BR, Oles A, Talbert EE, Romeo MJ, Vaena S, Sharma S, et al. Muscle inflammation is regulated by NF-kappaB from multiple cells to control distinct states of wasting in cancer cachexia. Cell Rep. 2024;43:114925. doi:10.1016/j.celrep.2024.114925

44.	Torres-Ruiz J, Alcala-Carmona B, Alejandre-Aguilar R, Gomez-Martin D. Inflammatory myopathies and beyond: The dual role of neutrophils in muscle damage and regeneration. Front Immunol. 2023;14:1113214. doi:10.3389/fimmu.2023.1113214

45.	Callaway CS, Delitto AE, Patel R, Nosacka RL, D'Lugos AC, Delitto D, et al. IL-8 Released from Human Pancreatic Cancer and Tumor-Associated Stromal Cells Signals through a CXCR2-ERK1/2 Axis to Induce Muscle Atrophy. Cancers (Basel). 2019;11:doi:10.3390/cancers11121863

46.	Land WG. The Role of Damage-Associated Molecular Patterns (DAMPs) in Human Diseases: Part II: DAMPs as diagnostics, prognostics and therapeutics in clinical medicine. Sultan Qaboos Univ Med J. 2015;15:e157-70.

47.	Luo YE, Villani KR, Lei H, Kuo LY, Imery I, Stoker BE, et al. Ablation of specific insulin-like growth factor I forms reveals the importance of cleavage for regenerative capacity and glycosylation for skeletal muscle storage. FASEB J. 2024;38:e23634. doi:10.1096/fj.202302512RR

48.	Lee YI, Cacciani N, Wen Y, Zhang X, Hedstrom Y, Thompson W, et al. Direct electrical stimulation impacts on neuromuscular junction morphology on both stimulated and unstimulated contralateral soleus. J Cachexia Sarcopenia Muscle. 2023;14:1533-45. doi:10.1002/jcsm.13235

49.	Lee YI, Li Y, Mikesh M, Smith I, Nave KA, Schwab MH, et al. Neuregulin1 displayed on motor axons regulates terminal Schwann cell-mediated synapse elimination at developing neuromuscular junctions. Proc Natl Acad Sci U S A. 2016;113:E479-87. doi:10.1073/pnas.1519156113
